# The RNA‐binding protein Snd1/Tudor‐SN regulates hypoxia‐responsive gene expression

**DOI:** 10.1096/fba.2022-00115

**Published:** 2023-02-21

**Authors:** Juha Saarikettu, Saara Lehmusvaara, Marko Pesu, Ilkka Junttila, Juha Partanen, Petra Sipilä, Matti Poutanen, Jie Yang, Teemu Haikarainen, Olli Silvennoinen

**Affiliations:** ^1^ Institute of Biotechnology, HiLIFE Helsinki Institute of Life Sciences University of Helsinki Helsinki Finland; ^2^ Faculty of Medicine and Health Technology Tampere University Tampere Finland; ^3^ Fimlab Laboratories Tampere University Hospital Tampere Finland; ^4^ Northern Finland Laboratory Centre (NordLab) Oulu Finland; ^5^ Research Unit of Biomedicine University of Oulu Oulu Finland; ^6^ Faculty of Biological and Environmental Sciences University of Helsinki Helsinki Finland; ^7^ Research Centre for Integrative Physiology and Pharmacology, and Turku Center for Disease Modeling, Institute of Biomedicine University of Turku Turku Finland; ^8^ Department of Immunology Tianjin Medical University Tianjin P.R. China

**Keywords:** BioID, Gene expression, Hypoxia, Knockout, Snd1, Stress‐reponse

## Abstract

Snd1 is an evolutionarily conserved RNA‐binding protein implicated in several regulatory processes in gene expression including activation of transcription, mRNA splicing, and microRNA decay. Here, we have investigated the outcome of *Snd1* gene deletion in the mouse. The knockout mice are viable showing no gross abnormalities apart from decreased fertility, organ and body size, and decreased number of myeloid cells concomitant with decreased expression of granule protein genes. Deletion of *Snd1* affected the expression of relatively small number of genes in spleen and liver. However, mRNA expression changes in the knockout mouse liver showed high similarity to expression profile in adaptation to hypoxia. MicroRNA expression in liver showed upregulation of the hypoxia‐induced microRNAs miR‐96 and ‐182. Similar to Snd1 deletion, mimics of miR‐96/182 enhanced hypoxia‐responsive reporter activity. To further elucidate the function of SND1, BioID biotin proximity ligation assay was performed in HEK‐293T cells to identify interacting proteins. Over 50% of the identified interactors were RNA‐binding proteins, including stress granule proteins. Taken together, our results show that in normal growth conditions, Snd1 is not a critical factor for mRNA transcription in the mouse, and describe a function for Snd1 in hypoxia adaptation through negatively regulating hypoxia‐related miRNAs and hypoxia‐induced transcription consistent with a role as stress response regulator.

## Graphical abstract

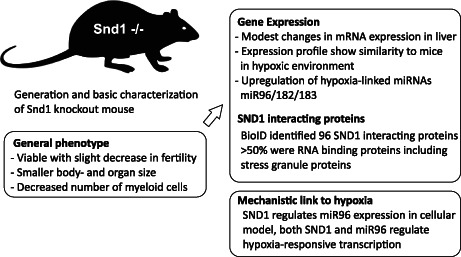

**Abbreviations**
BAC: bacterial artificial chromosome
Cas9: CRISPR associated protein 9
CRISPR: clustered regularly interspaced short palindromic repeats
DMOG: dimethyloxalylglycine
ELA: elastase
FACS: fluorescence‐activated cell sorting
FMR1: fragile X mental retardation 1
FPKM: fragments per thousand nucleotides per million mapped reads
G3BP1: Ras GTPase‐activating protein‐binding protein 1
HEK: human embryonic kidney
HIF: hypoxia‐inducible factor
IL: interleukin
KD: knockdown
KO: knockout
LCN: lipocalin
miRNA: MicroRNA
MPO: myeloperoxidase
MTDH: metadherin
qRT‐PCR: quantitative Real‐Time PCR
RISC: RNA‐induced silencing complex
RNAi: RNA interference
Serpin: serine protease inhibitor
ShRNA: short hairpin RNA
Snd1: Staphylococcal nuclease and Tudor domain containing 1
STAT: signal transducer and activator of transcription
TNF: tumor necrosis factor
UAS: upstream activating sequence
UTR: untranslated region
WT: wild type

## 1 INTRODUCTION

*Staphylococcal Nuclease and Tudor domain containing 1*, *Snd1* (*Tudor‐SN*, *p100*, *TDRD11*), is a phylogenetically conserved gene present predominantly as a single copy gene in all organisms from fission yeast (*Schizosaccharomyces pombe*) to human but not in bacteria or *Saccharomyces cerevisiae*. The protein consists of five staphylococcal nuclease‐like (SN) domain repeats and a C‐terminal Tudor‐domain that belongs to a family of methyl‐arginine and ‐lysine binding proteins implicated in regulation of RNA metabolism.^1^ The SN domains show similarity to *Staphylococcus aureus* Ca^2+^‐dependent extracellular nucleases and contain a conserved oligonucleotide‐binding scaffold.

Snd1 was initially identified as a transcriptional coactivator of the viral Epstein–Barr virus nuclear antigen 2 (EBNA‐2).^2^ Subsequently, the protein was shown to act as a coactivator for cellular transcription factors c‐Myb, STAT5, and STAT6^3^, ^4^, ^5^ that play important functions in signaling and transcriptional regulation of hematopoietic cells. The interaction with STAT transcription factors was shown to be mediated by the Snd1 SN‐domains. Snd1 has also been implicated in RNA interference through interaction with the RNA‐induced silencing complex (RISC)^6^ and in decay of a specific set of microRNAs (miRNAs).^7^ In a separate study, the *C. elegans* Snd1 was also shown to function as a calcium dependent nuclease with specificity to single‐ or double‐stranded RNA containing mismatched base pairs, such as A‐I edited RNA that has also previously been identified as Snd1 nuclease target.^8^, ^9^ Snd1 has been shown to affect pre‐mRNA splicing, as the Tudor domain binds dimethylated arginines in the U5 small nuclear ribonucleoproteins (snRNP) and facilitates spliceosomal assembly in vitro and the kinetics of the first stage of splicing.^10^, ^11^

Snd1 is broadly expressed in mammals with highest expression in dividing cells, epithelial cell types and organs with a secreting function, such as exocrine pancreas and lactating mammary gland, while muscle cells lack Snd1 expression.^12^, ^13^ Snd1 is overexpressed in several cancers including prostate, hepatic, and colon cancer and B cell malignancies,^14^ and several functional roles have been assigned for Snd1 in promoting tumorigenesis and cancer growth. Snd1 and the RNA‐binding protein Sam68, for example, co‐operate in regulation of alternative splicing to produce an oncogenic isoform of CD44 in prostate cancer.^15^ In addition, interaction of Snd1 with the RNA‐binding protein Metadherin (MTDH) was shown to be critical for early‐stage tumorigenesis in oncogene and carcinogen‐induced mammary tumor models where Snd1 stabilized the expression of pro‐survival genes under stress conditions.^16^ Snd1 is directly linked to cell proliferation through interaction with E2F‐1^17^ and through regulation of miRNA decay affecting the expression of cell‐cycle genes.^7^ Recently, Snd1 was suggested to promote hepatocellular carcinoma by binding and degrading the *Protein Tyrosine Phosphatase Nonreceptor Type 23* (*PTPN23*) mRNA.^18^ Snd1 is also a phylogenetically conserved caspase target implicated in programmed cell death.^19^

Several lines of evidence imply that Snd1 is involved in cellular stress responses. Snd1 is normally detected rather diffusely in cultured mammalian cells but relocates to stress granules in response to heat shock and arsenate treatment.^20^, ^21^ Similar relocation to RNA‐containing granules was demonstrated for the plant *Arabidopsis thaliana* Snd1/Tudor‐SN in response to heat stress.^22^ Stress granules are considered to protect from stress by storing mRNAs and stalling their translation, although experimental evidence that stress granules serve this protective function is still lacking. Genetic deletion of *Tudor‐SN* in *Arabidopsis* provides additional evidence for a role for *Tudor‐SN* in stress as the *Tudor‐SN* knockout plants have decreased survival in high salinity stress.^23^ In *Drosophila melanogaster*, *Tudor‐SN* deletion was shown to affect spermatogenesis and male fertility.^24^

Although in vitro experiments indicate a role for Snd1 in regulation of gene expression and RNA metabolism, the exact physiological functions of Snd1 in mammals are currently unclear. Here, we have generated and characterized the mouse knockout (KO) of *Snd1*. The null mice are viable and show no marked developmental or physiological defects in normal growth conditions, but present reduced fertility, body weight, and size. The knockout mice, furthermore, show lower numbers of mature macrophages and granulocytes that parallel with decreased expression of azurophilic granule protein encoding genes. The gene expression analysis of the KO liver shows expression alterations that closely correlate with expression changes in the liver of mice adapted to hypoxia.^25^ Our results support a mechanism for Snd1 in hypoxia by negatively regulating the expression of the hypoxia‐related microRNAs miR‐96‐5p, miR‐182‐5p. In summary, this work shows that *Snd1* is not essential for mouse development and survival in pathogen‐free environment, but the results show a role for *Snd1* in hypoxia response, possibly in adaptation to hypoxia, compatible with a physiological function as stress response regulator.

## 2 MATERIALS AND METHODS

### 2.1 Generation of the *Snd1* knockout mouse

**FIGURE 1:**
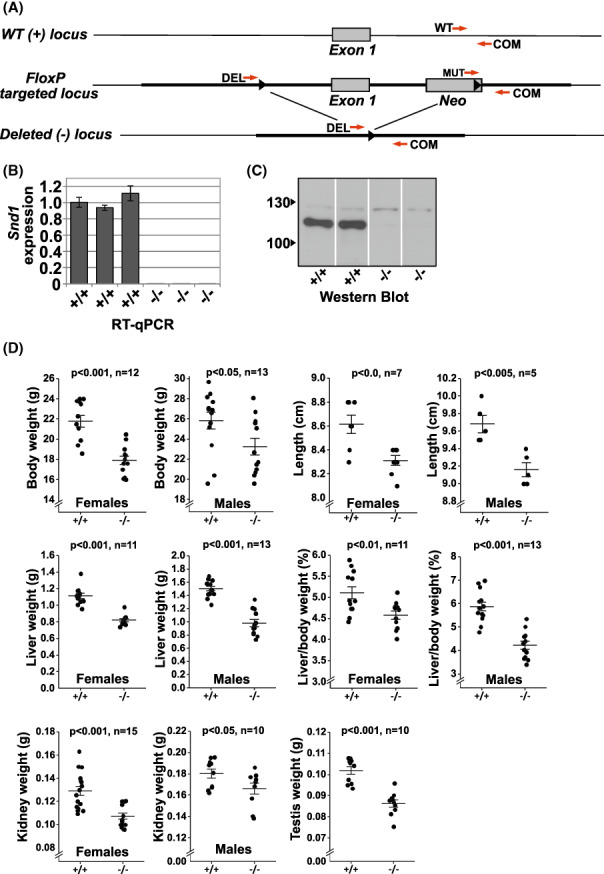
*Snd1* knockout mouse. (A) Schematic presentation of the *Snd1* gene targeting. Shown is the wild type locus (WT), *FloxP* targeted locus, and the locus after Cre‐mediated recombination (Deleted locus). Red arrows indicate primers used in PCR‐based genotyping. (B) Quantitative Real‐Time PCR of *Snd1* using RNA isolated from embryonic fibroblasts derived from wild type (+/+) and knockout (−/−) mice. (C) Western Blot analysis of Snd1 protein using embryonic fibroblasts derived from wild type and knockout mice. (D) Body weights of 2‐month‐old mice of indicated genotypes, nose to tail lengths, liver weights, body weight normalized liver weights, kidney weights of 2‐month old mice and testis weights of 2‐month old males. Littermates from offspring of heterozygous breeding pairs was used. Error bars indicate the standard error of the mean. The *p*‐values are calculated using unpaired two‐tailed *t*‐test and (*n*) indicates the number of animals of each genotype.

*Snd1* knockout mouse was generated by targeting the first exon, by placement of two *loxP* sequences to flank the exon (Figure 1A) and crossing these mice with Cre recombinase expressing mice. The first exon of the *Snd1* gene contains the sequences for 5′‐UTR and the first 26 amino acids of the protein. Approximately 10.3 kb‐long genomic sequence flanking the first exon was inserted by recombineering cloning technique^26^ from the BAC clone bMQ‐464N16 to the PL253 retrieval vector (the primers used in the cloning are given in the Table S1). The 5′ *loxP‐* and the 3′ l*oxP*‐sequences together with the Neo‐cassette were further inserted into the targeting vector using recombineering. The targeting vector, linearized with NotI, was then introduced into the R1 (129/Sv) mouse embryonic stem (ES) cells by electroporation, and stable clones were selected in the presence of 0.15 mg/mL G418. Southern Blot using probes specific for the sequences outside of the targeting construct was used to identify clones with homologous recombination. ES‐cells bearing the homologous targeting were used to generate chimeric mice in C57BL/6N background and the germline‐transmitted lineage at the Turku Center for Disease Modeling (TCDM). The *Snd1* FloxP mouse lineage was crossed with CAG‐Cre mice^27^ containing the Cre recombinase under control of the cytomegalovirus immediate early enhancer‐chicken β‐actin hybrid promoter. This created offspring with germline deletion of the first exon. The mice used in this study were the offspring after backcrossing the germline‐deleted mice to C57BL/6N for 6–10 generations. Littermates from heterozygous breeding pairs were used in the experiments. PCR genotyping of the mice was with the following primer pairs: CCCTGCAATGTGGTCAGGTCAT and AGCCTCCAGGGTATCTGGGTTC for the WT allele, product size 550 base pairs; CCCTGCAATGTGGTCAGGTCAT and CGCGTCGACGCATGTTCATGCTCCACGTA for the KO allele, product size 850 base pairs.

### 2.2 Flow cytometry

Spleen, thymus, and bone marrow cells of approximately 12‐week‐old male mice were collected and stained with antibodies for FACS analysis. Single‐cell suspensions were prepared in Phosphate‐Buffered Saline (PBS) by flushing the cells through 70 μm nylon mesh cell strainer (BD Biosciences, San Jose, CA). Red blood cells were removed from spleen and bone marrow using ACK‐lysing buffer (LONZA, Verviers, Belgium). Approximately 10^6^ cells were used for each FACS staining in PBS supplemented with 1% fetal bovine serum for 30 min at room temperature. Marker antibody dilutions were as follows: B220 PE (1:300), CD3 APC (1:300), CD11b PE‐CY7 (1:300), CD4 FITC (1:300), CD8 PERCP cy5.5 (1:300), Gr‐1 (Ly6G) PERCP cy5.5 (1:1000), and F4/80 APC (1:300). All the antibodies were from eBioscience (San Diego, CA). Annexin V (eBioscience) staining was performed according to manufacturer's instructions. Flow cytometry was performed using the Becton Dickinson FACSCanto II flow cytometer, and the data were analyzed using the FlowJO software (Becton, Dickinson and Company, Franklin Lakes, NJ).

### 2.3 RNA purification

For RNA extraction, half of the spleens of 8–12‐week‐old female mice were homogenized in 1 mL of Trizol reagent (Invitrogen, Carlsbad, CA) using the PowerLyzer homogenizer (MO BIO Laboratories, Carlsbad, CA) with ceramic beads. For RNA extraction of the livers, approximately 0.1 g piece of the left liver lobe of 8–12‐week‐old male mice were homogenized. The liver RNA samples were further purified using the DNA‐free kit (Ambion).

### 2.4 Gene expression analyses

For the spleen samples, biological duplicate RNA samples were used in hybridization to Illumina MouseRef‐8 v2.0 array (Illumina, San Diego, CA) using the standard protocol at the Institute for Molecular Medicine Finland (FIMM) Technology Centre Genomics Unit, University of Helsinki, and the probe signal intensities were obtained using the Illumina Genome Studio software. Liver array analysis was carried out with biological quadruplicate samples at the Yale center for genome analysis, Yale University, West Haven, CT, with Illumina MouseRef‐8 v2.0 array. The normalized intensity values were used in the 2‐group statistical test for differential expression analysis using the Chipster software (CSC – IT Center for Science, Espoo, Finland).

RNA‐seq analysis runs (small‐RNA‐ and mRNA‐seq) for mouse biological quadruplicate liver samples were carried out using the isolated total‐RNA at the Beijing Genomics Institute (BGI), Hong Kong: For small‐RNA seq analysis, the total‐RNA was polyacrylamide separated to obtain 18–30 base RNA fragments. Library preparation included 5′‐adenylated, 3′‐blocked single‐stranded DNA adaptor ligation to 3′ end of the RNAs, reverse primer annealing and cross‐linking to the 3′‐adaptor, and 5′‐adaptor ligation. The library was reverse transcribed, PCR amplified, and size separated using a polyacrylamide gel for the fragment size of 100–120 bp. The double stranded PCR products were heat denatured and circularized by the splint oligo sequence. The single strand circle DNA was formatted as the final library. The library was sequenced using the BGISEQ‐500 platform with single‐end reads of 50 bases. The impurities in the raw data were removed, including 5′ primer contaminants, no‐insert tags, oversized insertion tags, low quality tags, poly‐A tags, and small tags, and tags without 3′ primer. The Bowtie tool was used to map the reads to the reference genome and DEGseq for differential expression analysis of the miRNAs.

For mRNA sequencing of the mouse liver samples at BGI, the total‐RNA samples were purified using poly‐dT oligo attached magnetic beads. The mRNA was cleaved using elevated temperature and divalent cations and reverse transcribed to first strand cDNA followed by second strand cDNA synthesis using DNA polymerase I and RNAse H. The synthesized cDNA was subjected to end‐repair and then 3′ adenylated. Adapters were ligated to the ends of these 3′ adenylated cDNA fragments, and the fragments were PCR amplified. Samples were pooled together to make a single strand DNA circle (ssDNA circle), giving the final library. DNA nanoballs (DNBs) were generated with the ssDNA circle by rolling circle replication (RCR) to enlarge the fluorescent signals at the sequencing process. The DNBs were loaded into the patterned nanoarrays, and pair‐end reads of 100 bp were read through on the BGISEQ‐500 platform. The BGISEQ‐500 platform combines the DNA nanoball‐based nanoarrays and stepwise sequencing using the Combinational Probe‐Anchor Synthesis Sequencing Method. BGI internal software SOAPnuke was used to remove adaptor reads, reads that contain more than 5% unknown bases and low‐quality reads. Resulting reads in FASTQ format were aligned to the *Mus musculus* genome (genome assembly GRCm38.92) using the TopHat2 tool in the Chipster software. Quantification of reads per gene was performed using the HTseq tool. The EdgeR tool was used for differential expression analysis for the mouse liver. Cufflinks was used for retrieving the FPKM (fragments per thousand nucleotides per million mapped reads) values.

For quantitative Real‐Time PCR analysis (qRT‐PCR), total RNA (4 μg) was reverse transcribed using M‐MuLV reverse transcriptase (Thermo Fisher Scientific) according to manufacturer instructions. The cDNAs were diluted 1:10 in water, and two μL of this was used in 10 μL qRT‐PCR reaction using Maxima SYBR Green qPCR Master Mix (Thermo Fisher Scientific) and primers at a concentration of 0.3 pmol/μL. List of the qPCR primers is given in the Table S2. qRT‐PCRs were performed using the Bio‐Rad CFX‐96 or CFX‐384 Real‐Time PCR detection system. Starting quantity of each gene's transcript was determined by fitting the qPCR data to a standard curve, and the expression of each gene was normalized to the expression of *TATA‐box binding protein* (*Tbp*) for the mouse genes. The miRCURY LNA miRNA PCR system (Qiagen) including the reverse transcription reagent and the SYBR green polymerase were used for qRT‐PCR analyses of the microRNA expression. MiRCURY LNA miRNA PCR primers hsa‐miR‐96‐5p, hsa‐miR‐182‐5p, and the U6 snoRNA for normalization were also from Qiagen. Western blotting was carried out as previously described using the anti‐SN4‐1 antibody to detect the mouse Snd1 protein.^28^

### 2.5 Cell culture, expression vectors, and transfections

The HEK‐293T (Human Embryonic Kidney) cells were maintained in humidified 37°C, 5% CO_2_ incubator in Dulbecco's modified Eagle's medium (DMEM) supplemented with 10% fetal bovine serum and antibiotics. The SND1 CRISPR‐Cas9 targeting construct was generated using a previously described approach.^29^ The oligonucleotide dimers used in generating the SND1 and control specific guide RNAs have been described previously.^30^

The HRE‐LUC vector was cloned by inserting a concatemer containing eight copies of oligonucleotide dimers (GATCTCCCGCGGCGTACGTGCCGGGCGGCACGGCCG and GATCCGGCCGTGCCGCCCGGCACGTACGCCGCGGGA) with HIF‐1α binding consensus DNA‐sequence into the BglII site in the Luciferase reporter vector pGL 4.23 (Promega, Madison, WI) that contains a minimal basal promoter downstream of the cloning site. For Luciferase assay transfections, HEK‐293T cells plated at 50 000 cells per well on 24‐well plates were co‐transfected with 50 ng of HRE‐LUC and 50 ng of CMV‐βGAL vector using the JetOPTIMUS transfection reagent. 24 h after transfection, hypoxia condition was induced by addition of 0.5 or 1.0 mM DMOG (Dimethyloxalylglycine, EMD Millipore). DMOG induction was for three days, and the cells were harvested for luciferase assay performed with the Luciferase Assay System (Promega) and colorimetric β‐galactosidase assay using ONPG (o‐Nitrophenyl‐β‐galactoside) as the substrate. MicroRNA mimics MISSION microRNA hsa‐miR‐96, hsa‐miR‐182, and the Negative Control miRNA 1 were all from Sigma‐Aldrich. MicroRNA mimics were transfected using INTERFERin transfection reagent (Polyplus Transfection). Two nM of microRNA mimic was used in the transfections. The transfection complexes of expression vectors (HRE‐LUC and CMV‐βGAL) with JetOPTIMUS were mixed with the microRNA mimic‐INTERFERin complexes for performing the co‐transfection.

### 2.6 BioID biotin proximity ligation assay

To construct the Snd1‐BioID2 fusion, the full‐length mouse *Snd1* cDNA (the mouse and human Snd1 sequence are 98% identical at the protein level) was PCR amplified using the primers CTCTACCGGTGCCATGGCCTCCTCCGCGCAGAGCAG (forward) and CTCTGGATCCGCGACTGTAGCCAAACTCATCAG (reverse) and cloned into *AgeI* and *BamHI* sites in the vector pcDNA3.1 MCS‐BioID2‐HA (a gift from Kyle Roux, Addgene plasmid #74224) to produce pcDNA3.1 Snd1‐BioID2‐HA. HEK‐293T cells were grown in DMEM medium supplemented with 10% fetal bovine serum and antibiotics and maintained at 37°C in a humidified incubator containing 5% CO_2_. For large‐scale transient transfections, 80%–90% confluent cells grown in 15 cm diameter plates were transfected using linear polyethyleneimine^31^ with 20 μg of plasmid DNA, either pcDNA3.1 Snd1‐BioID2‐HA or control pcDNA3.1 MCS‐BioID2‐HA. Biotin (Sigma‐Aldrich B4639) was added 24 h after transfection to 10 μM concentration. For the cells to be treated with heat shock, Hepes‐KOH pH 7.3 was added to 20 mM final concentration with 125 mM NaCl in the added volume. Heat shock was for 12 h at 42°C in an incubator without added CO_2_ gas. For hypoxia treatment, the transfected cells were placed at 2% oxygen 5% CO_2_ 37°C incubator for 12 h. The cells were rinsed twice with PBS, collected by centrifugation and lysed in 20 cell pellet volumes of RIPA‐500 lysis buffer (50 mM Tris–HCl pH 7.5; 500 mM NaCl; 1% Triton X‐100; 0.2% SDS; 0.5% sodium deoxycholate; 1 mM EDTA, and 1 mM DTT) supplemented with protease inhibitors (1 mM PMSF; 4 μg/mL aprotinin and 4 μg/mL pepstatin). The cell lysate was sonicated and after centrifugation at 12,000*g*, 30 min further cleared by filtering through a 45 μm filter. Biotinylated proteins were captured overnight to 50 μL of streptavidin‐agarose beads (Sigma‐Aldrich S1638). The beads were washed 5 times with 1 mL of RIPA‐500 buffer with 5 min rotation of the tubes between washes followed by 4 × 1 mL washes with 50 mM Tris pH 8.0. The samples were on‐beads digested at the Turku Proteomics Facility, Turku Centre for Biotechnology according to a standard protocol and analyzed by LC‐ESI‐MS/MS using a Q Exactive mass spectrometer. Database searches were performed by Mascot 2.6.1 against SwissProt protein sequence database.

### 2.7 Ethical approval for animal experimentation

The work is approved by the ethical committee of the District of Southern Finland (ESAVI/9580/04.10.07/2014).

## 3 RESULTS

### 3.1 Reduced body weight and size in *Snd1* KO mice

Based on cell culture studies, several functions have been assigned for Snd1, most of which relate to regulation of gene expression. In order to address the question of Snd1 function in vivo, we carried out the gene deletion in mouse. *Snd1* exists in mammals as a single copy gene with no close homologs; thus, the knockout should give reliable results on the physiological functions of the gene. The KO mouse was generated by deleting the first exon of the gene. qRT‐PCR and western blot analysis showed the absence of Snd1 protein in cell and tissue extracts from homozygous KO mice (Figure 1B,C). The loss of Snd1 protein was confirmed with two independent antibodies (against the SN4 and Tudor domain). The homozygous KO mice were viable showing no obvious effect on the well‐being or physical activity. Both the male and female homozygous KO mice were fertile, but the average litter size from KO breeding pairs was less than 50% of the litter size from WT breeding pairs (average 4 pups vs. 9). From heterozygous breeding pairs, the homozygous knockouts were born at approximately 35% lower frequency than expected. *Snd1* KO mice had modest, but statistically significant decrease in body weight, as shown for 2 months‐old animals in Figure 1D. The weight difference was not significant at the age of 1 week after birth but increased during the growth of the mice (Figure S1). An approximately 15%–20% decrease (*p* < 0.05) in the weight of the KO animals was observed still at the age of 6‐months. The lengths of the KO animals were also decreased (Figure 1D). Out of the major organs, liver showed most prominent decrease in weight that was evident also when normalized to body weight. The weights of testis and kidney were also decreased (Figure 1D), whereas no significant decrease was observed in weights of pancreas, spleen, muscle (thigh), lung, and ovary (data not shown). The weight and size phenotypes were evident in the knockout mice of both sexes. No apparent changes were observed in histological analysis of the KO mice, including the intestine, liver, testis, and kidney (Figure S2).

### 3.2 Myeloid lineage maturation is affected in *Snd1* KO mice

**FIGURE 2:**
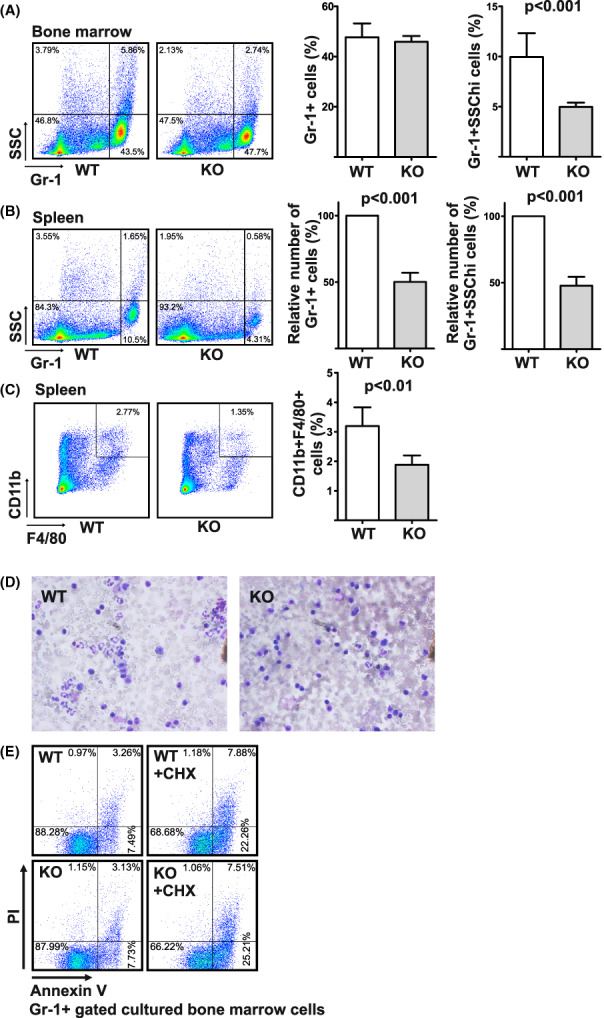
Myeloid cell deficiency in mice lacking *Snd1*. (A) Flow cytometric analysis of bone marrow Gr‐1+ granulocytes (left panel) and quantification of the numbers of Gr‐1+ granulocytes in bone marrow, and their granularity (*n* = 8, right panel). 12‐week‐old male mice were used in the analyses. Error bars indicate the standard error of the mean and the *p*‐values are calculated using two‐tailed Student's *t*‐test. (B) Flow cytometric analysis of spleen Gr‐1+ granulocytes (left panel) and quantification of Gr‐1+ granulocyte numbers in spleen and quantification of highly granular Gr‐1+ cells (*n* = 8, right panel). The quantification of spleen granulocytes was carried out as a pair‐wise comparison of the cell numbers within individual experiments, the number of WT cells set as 100%. (C) Flow cytometric analysis of the numbers of spleen CD11b+/F4/80+ mature macrophages (left panel) and quantification of the spleen mature macrophages (*n* = 8, right panel). (D) May‐Grünwald Giemsa staining of blood leukocytes. Blood samples were treated with red blood cell lysis buffer (10 mM Tris–HCl, pH 7.4, 8.3 g/L NH_4_Cl, 1 mM EDTA) followed by Cytospin centrifugation to microscope slides and May‐Grünwald Giemsa staining. (E) Apoptosis is not significantly affected in cultured granulocytes of Snd1 KO. Bone marrow cells from WT and Snd1 KO mice were cultured for 2 h in RPMI medium supplemented with 10% fetal bovine serum, in the presence or absence of 10 μg/mL Cycloheximide (CHX) for increased apoptosis. Flow cytometric analysis was carried out for Gr‐1, Annexin V and Propidium Iodide (PI) stained cells. Early apoptotic cells are Annexin V‐positive and PI‐negative, whereas late apoptic/necrotic cells are positive for both Annexin V and PI.

Snd1 has been implicated to serve as a coactivator for several transcription factors involved in hematopoiesis such as c‐MYB, STAT5, and STAT6. FACS analysis was carried out to analyze the hematopoietic cells in the KO mouse. The lymphocyte populations showed no differences between the KO and WT mice in terms of the absolute numbers and ratios between CD4 and CD8 positive T‐cells in thymus and spleen, and numbers and ratios between CD3 and B220 positive T‐ and B‐cells in spleen (Figure S3). In vitro expanded T‐cells from spleen (anti‐CD3 + anti‐CD28 crosslinking) showed normal secretion profiles for the major cytokines involved in T‐cell polarization (IL‐2, IL‐4, IL‐6, IL‐10, IFN‐γ, TNF, and IL‐17A) and bone marrow derived macrophages from WT and KO mice responded similarly to LPS by inducing expression of interleukin‐6 and TNF (Figures S4 and S5). Colony‐forming cell assays demonstrated equal numbers and proportions of progenitors differentiating to macrophage, granulocyte, and erythrocyte colonies from cells isolated from the WT and KO bone marrows (Figure S6). However, closer analysis of the bone marrow myeloid compartment in the mice revealed an overall decrease in granularity (SSC) of the Gr‐1 (Ly‐6G) marker positive granulocytes (Figure 2A). The number of the Gr‐1 positive granulocytes was lower in the KO spleens, and the cellular granularity was also significantly reduced in these spleens (Figure 2B). This myeloid cell phenotype was observed in both males and females. In accordance with the FACS quantification, May‐Grünwald Giemsa staining of blood smears showed a clear decrease in the number of granulocytes in leukocyte‐enriched blood samples in the KO as compared to WT mice (Figure 2D). Similarly, the number of mature macrophages expressing CD11b and F4/80 was lower in the KO spleens (Figure 2C). The decrease in the number of granulocytes in the KO is not likely to result from increased apoptosis since cultured granulocytes did not show altered sensitivity to apoptosis in response to cycloheximide treatment (Figure 2E).

### 3.3 Gene expression in spleen

**FIGURE 3:**
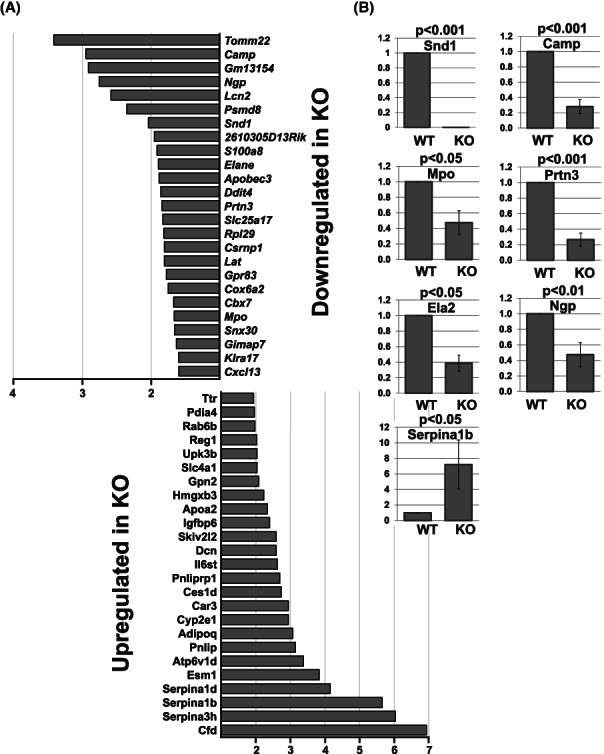
Differential gene expression in spleen of the *Snd1* knockout mice. (A) Duplicate RNA samples of WT and KO spleens from 8‐week‐old female mice were used in Illumina bead array gene expression analysis. The average fold‐change in gene expression of the most highly affected genes is shown. (B) Validation of the differential expression analysis. The average relative expression of *Snd1*, *Camp*, *Mpo*, *Prtn3*, *Ela2*, *Ngp* and *Serpina1b* in spleens in WT and KO mice was assessed by qRT‐PCR. 8–12‐week‐old female mice (6 WT and 6 KO) were used. The level of expression of each gene in WT mice was set to 1. Error bars indicate the standard error of the mean. The *p*‐values are calculated using paired two‐tailed *t*‐test.

Snd1 has been shown to function as transcriptional coactivator, and it was of interest to determine the effect of *Snd1* deletion on global gene expression in vivo. The gene expression profile using microarray was first analyzed in spleen, which reflects the peripheral hematopoietic milieu. A relatively small number of genes showed altered expression in the KO spleens. Approximately 50% reduced expression was observed for genes encoding for neutrophil granule proteins, including *Neutrophilic granule protein* (*Ngp*) and *myeloperoxidase* (*Mpo*) (Figure 3A). In addition, neutrophil‐expressed serine proteases *Neutrophil elastase* (*Ela2*/*Elane*) and *proteinase 3* (*Prtn3*) were downregulated. The differential expression of these genes was validated using qRT‐PCR (Figure 3B). The downregulation of myeloid‐cell specific transcripts in spleen could reflect the decreased number of myeloid cell types in the spleen (Figure 2). Interestingly, the serine protease inhibitors of the *Serpina* family were highly over‐expressed in KO mice. Thus, Snd1 has opposite effect on expression of serine proteases and their functional antagonists, serine protease inhibitors that may have relevance in regulation of the inflammatory response in vivo. To this end, we analyzed if Snd1 can directly regulate the transcription of the abovementioned myeloid genes by using shRNA knockdown. Silencing of *SND1* in K562 proerythroblastic cells did not affect the promoter activities of *ELA2*/*ELANE*, *MPO* or *LCN2* analyzed by luciferase reporter assays (Figure S7) and silencing of *SND1* in HL‐60 promyelocytic cells did not affect the expression of endogenous *ELA2* and *MPO* genes (data not shown). These data suggest that Snd1 does not directly regulate the promoter activities of the myeloid cell genes.

### 3.4 Gene expression in *Snd1* KO liver with hypoxia‐specific expression changes

**FIGURE 4:**
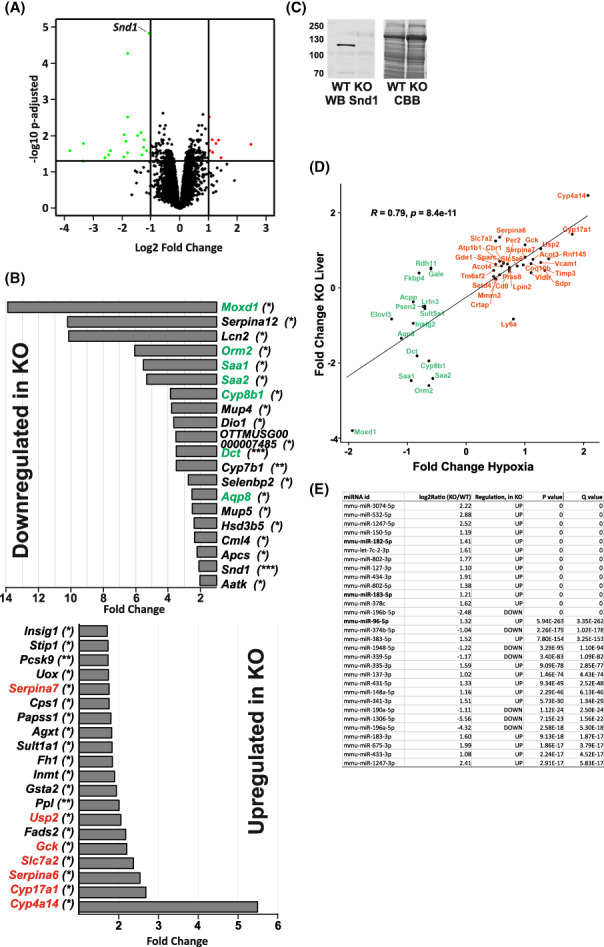
Differential gene expression in liver of the *Snd1* knockout mice and comparison to gene expression changes in hypoxic environment. (A) Volcano plot of differential gene expression in Snd1 KO liver. Quadruplicate RNA samples of WT and KO livers from 8–12‐week‐old male mice were used in Illumina bead array gene expression analysis. (B) Average fold‐change in gene expression of the most highly affected genes. Statistical significance (*p*‐adjusted) is indicated in the parenthesis (*padj < 0.05, **padj < 0.005, ***padj < 0.001). Genes indicated by green and red color were found similarly deregulated in mouse livers in response to simulated high‐altitude hypoxic environment^25^ (green for downregulated and red for upregulated genes) (C) Western Blot (WB) detection of Snd1 protein in extracts from WT and KO livers. Duplicate samples were Coomassie Brilliant Blue stained (CBB) for total protein levels (D) Correlation analysis of genes differentially expressed in *Snd1* KO liver and in high‐altitude hypoxic condition. Figure shows the common deregulated genes (*p* < 0.05) in the expression analyses of WT vs. *Snd1* KO liver and altitude of 1400 m vs. simulated altitude of 4500 m. Fold changes are presented as log2 values. (E) Small RNA‐seq analysis of differentially expressed miRNAs in the *Snd1* KO liver. Quadruplicate RNA samples of WT and KO livers from 8–12‐week‐old male mice were used in the analysis. Listed are the most significantly differentially expressed miRNAs that includes the hypoxia‐responsive miRNA cluster miR‐96, −182 and − 183.

Snd1 KO mice have reduced liver size. We wanted to analyze the effect of Snd1 deletion on gene expression in the organ and conducted a genome‐wide microarray gene expression analysis of livers of young adult KO and WT mice (Figure 4). Relatively small number of genes showed significantly altered expression in the KO (20 downregulated and 8 upregulated with over 2‐fold change in expression, *p* < 0.05, Figure 4A,B) supporting a view that Snd1 is not a global regulator of gene expression. Expression of *Snd1* itself was relatively low in the WT liver and therefore did not show among the highest downregulated genes in the analysis. Snd1 protein was nevertheless clearly detected by the western blot analysis of the WT liver and absent in the KO liver (Figure 4C).

To validate the gene expression data obtained by microarray analysis, we performed mRNA sequencing analysis of KO mouse liver samples. The sequencing data showed highly significant expression correlation with the array data (*R* = 0.88, *p* < 2.2 × 10^−16^) for 101 genes whose expression was significantly altered in KO mouse (Table S3 and Figure S8). RNA‐seq analysis also indicated *Snd1* as the most significant differentially expressed gene between WT and KO mice (Table S3).

Among the highest downregulated genes in both the microarray and RNA sequencing analysis were *Moxd1*, a monooxygenase with yet unknown function, and *Lipocalin 2* (*Lcn2*), *Orosomucoid 2* (*Orm2*), *Serum Amyloid A1*, and *‐2* (*Saa1* and *‐2*) that all encode major acute phase proteins. To test the general involvement of Snd1 in inflammation, we focused on interleukin‐6 and Stat3 that are central regulators of acute‐phase proteins expression. However, LPS triggered induction of interleukin‐6 expression was similar in *Snd1* KO and WT macrophages (Figure S5). Furthermore, *Snd1* silencing did not affect STAT3‐driven luciferase reporter activity (data not shown) suggesting that the effect of Snd1 on acute phase gene expression is not through direct transcriptional regulation.

The gene expression pattern in the *Snd1* KO liver was compared to expression analyses of mouse livers available at the Gene Expression Omnibus (GEO) repository (https://www.ncbi.nlm.nih.gov/geoprofiles). We found a striking correlation between the differentially expressed genes in *Snd1* KO liver and gene expression changes in adaptation to simulated high‐altitude (4500 m) hypoxic environment^25^ (expression data accessible at the NCBI GEO database, accession GSE15891) regarding both downregulated and upregulated genes (Figure 4B,D). RNAseq data analysis further confirmed the altered expression of the genes and their close correlation between KO mouse liver and hypoxic conditions (*R* = 0.78, *p* = 3.3 × 10^−7^, Figure S8).

### 3.5 SND1 regulates microRNAs in hypoxia response

To analyze if the role of Snd1 in hypoxia‐response could be mediated through regulation of microRNA expression, we carried out small‐RNA‐seq analysis in *Snd1* WT and KO livers (Figure 4E). A general overexpression of miRNAs was observed in the *Snd1* KO liver, which could correspond to the role of Snd1 in miRNA degradation.^7^ Among the overexpressed miRNAs, miR‐96, ‐182, and‐183 have been shown to regulate HIF1‐α by increasing protein stability.^32^, ^33^

**FIGURE 5:**
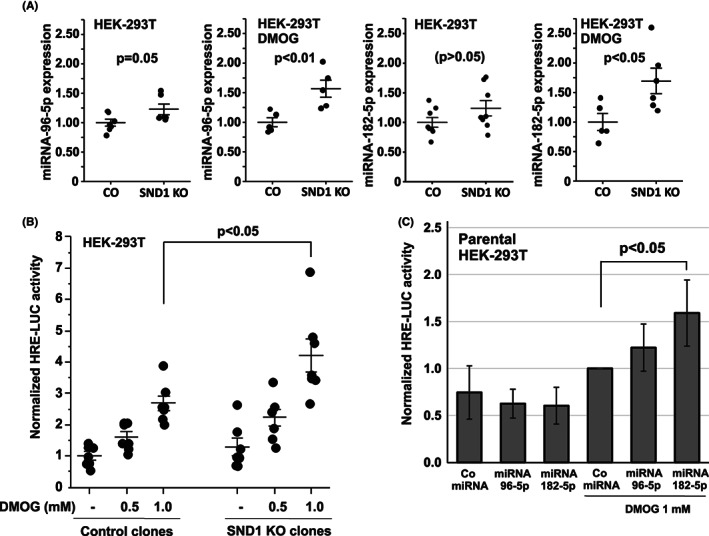
Mechanistic link between SND1, microRNAs miR‐96, miR‐182 and hypoxia (A) Expression levels of miR‐96‐5p and miR‐182‐5p in HEK‐293T SND1 KO cell clones and control clones (CO) in normal growth condition or in the presence of 1 mM DMOG. The values are from three independent experiments using 6–8 different WT and KO cell line clones. The horizontal line indicates the mean miRNA expression in the clones and the average expression of the CO clones is set as 1. miRNA expression was determined using qRT‐PCR analysis by normalizing to the U6 SnoRNA expression. The *p*‐values are calculated using paired two‐tailed *t*‐test. (B) The SND1 KO and control HEK‐293T cells were transfected with the HIF‐responsive HRE‐LUC reporter and treated, where indicated, with the HIF hydroxylase inhibitor DMOG (0.5 and 1.0 mM) to induce hypoxia‐mimicking conditions. The cells were harvested after three days of DMOG treatment to mimic chronic hypoxia and assayed for normalized luciferase activity. The data points are the average for each clone from four independent transfections and the horizontal lines indicate the mean and the standard error of the normalized activities of the clones. The average activity of the control clones in the absence of DMOG is set as 1. (C) HEK‐293T cells were transfected with the indicated microRNA mimics together with the HRE‐LUC reporter. The cells were treated, where indicated, with 1 mM DMOG for three days and harvested for analysis. The bars indicate the average normalized reporter activity from four independent transfections and the error bars indicate the standard deviation. The average activity of the control microRNA mimic in the presence of DMOG is set as 1.

Since the microRNAs miR‐96, miR‐182, and miR‐183 were upregulated in the Snd1 KO livers and have been shown to be also upregulated in the HEK‐293T cells upon SND1 knockdown,^7^ we wanted to analyze if SND1 knockout and the microRNAs affect hypoxia response in cells. SND1 knockout cell lines generated by CRISPR‐Cas9 technique were used in these experiments.^30^ First, we validated the miRNA upregulation in the SND1 KO cells using qPCR analysis. Both miR‐96‐5p and 182‐5p were slightly upregulated in the SND1 KO HEK‐293T cells, but treatment with the hypoxia‐inducing compound DMOG enhanced their expression in the KO cells to reach statistical significance in comparison to control cells (Figure 5A) (the expression of miR‐183‐5p was not analyzed as there was no commercial LNA qPCR primer available for the miRNA). Next, we analyzed the effect of SND1 KO on HIF‐dependent transcription using a HIF‐responsive HRE‐LUC luciferase reporter in a 3‐day induction with DMOG to mimic chronic hypoxia. Knockout of SND1 resulted in an increase in the activity of the hypoxia‐responsive reporter that was evident with the higher 1 mM concentration of DMOG (Figure 5B). Similarly to SND1 KO, transfection of miRNA mimics of miR‐96‐5p and miR‐182‐5p increased the HRE‐LUC reporter activity in the presence of DMOG (Figure 5C). These results suggest that the effect of SND1 on hypoxia could be through negatively regulating the expression levels of hypoxia promoting miRNAs miR 96‐5p and 182‐5p.

### 3.6 Identification of Snd1 interacting proteins

**FIGURE 6:**
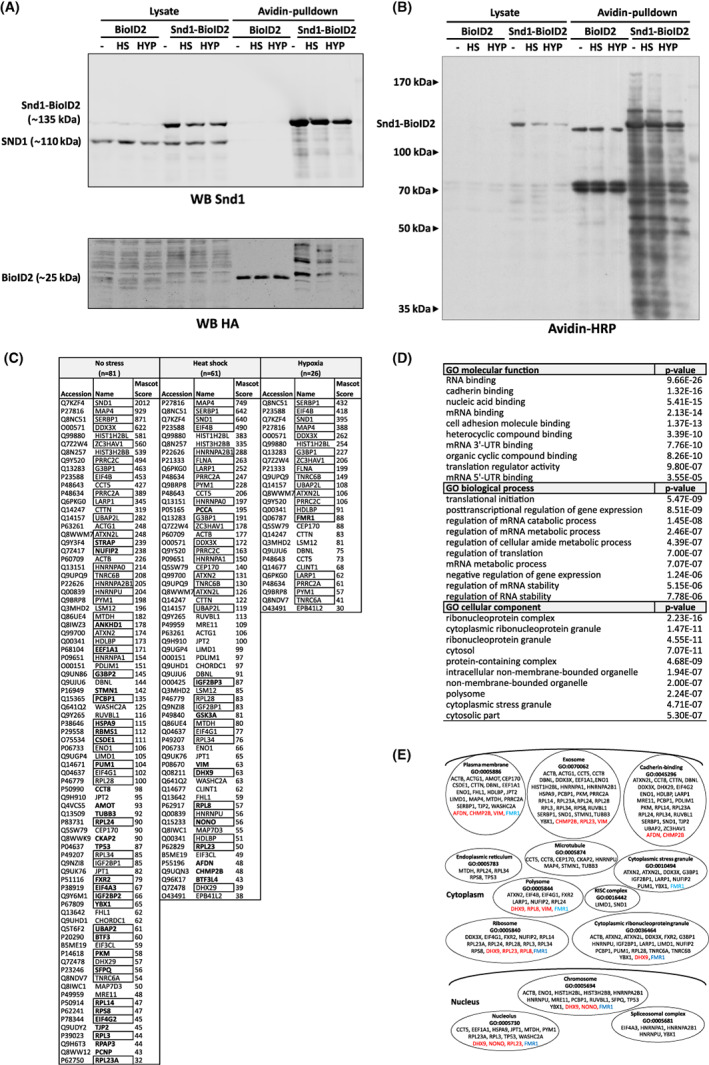
BioID analysis of Snd1 interacting proteins. (A) Western Blot analysis of HEK‐293T cells transiently transfected with either BioID2 or Snd1‐BioID2 expression vector and grown in either non‐stress condition (−) or 12 h of heat shock (HS, 42°C) or hypoxia (HYP, 2% oxygen). Lysates and biotinylated proteins captured with Avidin‐beads (Avidin‐pulldown) were resolved on SDS‐PAGE and blotted. Snd1‐BioID2 and the endogenous SND1 was detected with a Snd1 antibody (upper panel) and BioID2 with an antibody against Hemagglutinin A tag (HA, lower panel) (B) Detection of biotinylated proteins in the lysates and Avidin pulldown samples. Biotinylated proteins were detected with horseradish peroxidase –coupled Avidin (C) List of the identified Snd1 interacting/proximal proteins after Avidin‐pulldown and mass‐spectrometric identification. Listed are the identified proteins from non‐stressed growth condition, heat shock and hypoxia. The proteins that were identified also in the samples with empty BioID2 transfection are excluded from the list. The Mascot Score is relative to the number of identified peptides matching the protein. The proteins in the gene ontology ‐category of RNA‐binding proteins (GO: 0003723) are boxed and the proteins that are unique in each condition (non‐stressed, heat shock or hypoxia) are in bold (D) Gene ontology analysis of the identified putative Snd1 interacting proteins. PANTHER Overrepresentation Test (released 20181010) was used with GO Ontology database released 2018‐09‐06 with Fisher's Exact test with Bonferroni correction. (E) Visual presentation of the identified putative Snd1 interacting proteins sorted in cellular bodies and protein complexes. Proteins written in red were identified exclusively in heat shock and the protein in blue (FMR1) in hypoxia.

In order to obtain mechanistic insights to Snd1 function in stress responses, we used the Biotin Proximity Ligation Assay developed by Roux et al.^34^ to identify Snd1 interaction in protein complexes. Snd1 interacting proteins were analyzed in normal non‐stressed condition, in heat shock, and in hypoxia in HEK‐293T cells. HEK‐293T cells were chosen since they are easily transfected and widely used. Snd1‐BioID2 fusion construct consisted of the full‐length mouse *Snd1* fused to C‐terminal BioID2, a biotin ligase from *Aquifex aeolicus* that is smaller and requires less biotin as compared to the original BioID BirA biotin ligase.^35^ Transient transfection conditions for Snd1‐BioID2 were chosen to correspond to the endogenous expression level of Snd1 as assessed by immunoblotting (Figure 6A). Ninety‐six interacting proteins were identified that included the previously reported Snd1 interacting proteins G3BP1 and MTDH (Metadherin). Over 50% of the identified proteins were RNA‐binding proteins. The proteins with highest number of identified peptides, suggestive for abundant interaction or proximity with Snd1, were Snd1 itself, MAP4, SERBP1, EIF4B, DDX3X, and several histone variants (Figure 6C). The list of the putative interactors was not significantly affected by the heat shock and hypoxia stress conditions, except that the number of identified interactors was smaller in the stress conditions. The multifunctional RNA‐binding protein FMR1 was the only protein identified uniquely in hypoxia. The interactors were most highly enriched in the gene ontology categories of mRNA 5′‐ and 3′‐UTR‐binding, initiation of translation, cytoplasmic stress granule, and cadherin biding (Figure 6D). Cytoplasmic stress granule proteins (listed in Figure 6E) were identified also in non‐stressed conditions suggesting that Snd1 interacts with stress granule proteins also in physiological conditions. This finding is in accordance with results that stress conditions do not significantly affect interactions between stress granule proteins.^36^ These results support the proposed role for Snd1 in stress responses that is mediated through interactions with RNA and protein components in different ribonucleoprotein complexes.

## 4 DISCUSSION

Here, we describe the generation and characterization of the mammalian knockout model of *Snd1* that allows the analysis of the physiological function of this multifunctional RNA‐binding protein. The results show that Snd1 is not a critical factor for mouse development and survival in normal laboratory conditions, but the KO mice show reduction in size, decreased fertility, a specific effect on myeloid cells with decreased granularity and numbers of mature myeloid cells, and gene expression changes in liver that relate to hypoxia response. *Snd1* gene is highly conserved in species from *S. pombe* to human and exists in animals as a single copy gene without close homologs. Thus, the mouse KO phenotypes are not likely influenced by functional redundancy within a gene family and is expected to reflect the “true” function of the gene.

The *Snd1* gene homolog deletions have been generated in some non‐mammalian species. In the African trypanosome, deletion of *Snd1* did not affect the RNAi pathway, cell growth or differentiation,^37^ while deletion of the *Snd1* homologs in the plant *Arabidopsis thaliana* resulted in reduced root growth and fertility and decreased tolerance to stress conditions, which correlated with destabilization of certain mRNAs required for stress tolerance.^23^ The *Drosophila Tudor‐SN* has been linked to spermatogenesis and transposon silencing through interaction with the Piwi protein.^24^ Our histopathological analysis did not reveal apparent changes in the testis morphology or sperm development in the KO (Figure S2). It is thus likely that Snd1 has only a modulatory role in spermatogenesis, at least in mouse, through interactions with factors such as the Piwi homologs and the RNA‐binding protein Sam68 implicated in regulation of spermatogenesis.^15^

The spleens of *Snd1* KO mice had reduced number and granularity of mature granulocytes and monocytes that coincided with reduced expression of azurophilic granule genes (Figure 3). The downregulated expression of the serine proteases *Ela2* and *Prtn3* and upregulation of their functional antagonists, *Serpins*, in the KO mice suggests a role for Snd1 in orchestration of defense mechanisms, where the opposite gene responses converge functionally to strengthen the inflammatory response. One possibility for the observed myeloid phenotype would be the Snd1‐mediated regulation of transcription factors (coactivation or repression) that guide the timely expression of granule proteins. However, this we find unlikely since silencing or overexpression of *Snd1* in the human K562 cell line did not affect the activity of *Ela2‐*, *Lcn2‐*, *and Mpo‐*promoter driven reporter genes (Figure S7). These data indicate that Snd1 is not transcriptionally regulating the expression of myeloid genes.

*Snd1* KO livers were significantly smaller than in WT littermates (Figure 1D). However, the analysis of the *Snd1* KO liver mRNA transcriptome using microarray analysis showed relatively modest overall changes in gene expression. Only 28 genes were differentially expressed by over 2‐fold (Figure 4A,B). Transcripts encoding for acute‐phase plasma proteins, *Lcn2*, *Orm2*, *Saa1* and *Saa2* were among the most downregulated genes in the KO livers (Figure 4B). The microarray analysis was further supported by RNA‐seq analysis that showed similar results, including downregulation of the genes encoding acute‐phase plasma proteins. The acute‐phase genes are normally expressed at low levels and highly induced in inflammation. Snd1 could have a role in the steady‐state low level expression of the genes whereas the possible role in inflammation‐induced expression remains to be investigated. Interestingly and in accordance with our KO model, transgenic overexpression of *Snd1* in mouse liver has been shown to result in chronic inflammatory state with partial penetrance to hepatocellular carcinoma.^38^

The most striking finding of the gene expression analysis was the close correlation of the differentially regulated genes in the *Snd1* KO mice and mice exposed to long‐term hypoxic environment^25^ (Figure 4B,D). Response to low oxygen in hypoxic environment is a conserved function throughout the eukaryotic phylogeny. In vertebrates, hypoxia leads to stabilization of the Hypoxia Inducible Factors, HIF‐1a, ‐2a, and ‐3a. These transcription factors induce expression of genes to counteract the hypoxic challenge, such as the genes involved in glucose transport, glycolytic pathway and generation of new blood vessels. Silencing of *Snd1* in human HEK‐293T cells has been shown to result in increased expression of 35 miRNAs,^7^ of which four miRNAs are in this study found to be overexpressed also in the mouse *Snd1* KO liver. Interestingly, three out of the four common upregulated miRNAs (miR‐96‐5p, miR‐182‐5p, and miR‐183‐5p) that are conserved between mouse and human are shown to positively regulate the hypoxia response.^32^

We confirm by qPCR analysis the observed in vivo effect of SND1 loss on miR‐96‐5p and miR‐182‐5p expression in HEK‐293T cells (Figure 5A). The link between SND1 and the miRNAs in hypoxia is further supported by stimulations of hypoxic condition by DMOG treatment where both SND1 KO and overexpression of miR‐96‐5p/miR‐182‐5p mimic have similar stimulatory effect on the HRE‐LUC hypoxia reporter. It should be noted, however, that we did not observe any significant effect by SND1 KO on HIF1‐α stability or HRE‐LUC activity in short‐term (acute) inductions of hypoxia, but the effect was only observed when chronic hypoxia was simulated by DMOG treatment for several days. Therefore, we speculate that SND1 could be required for controlling adaptation to hypoxia rather than in the immediate hypoxia response. We also found that recombinant SND1 protein could bind the miR‐96‐5p and miR‐182‐5p RNAs in an Electrophoretic Mobility Shift assay (EMSA) (Figure S9) but could not detect any decay of the miRNAs by SND1 in the assay suggesting that the role of SND1 may be indirect in negative regulation of the miRNAs' expression rather than through direct degradation by SND1 nucleolytic activity. SND1 has also been previously shown to be involved in hypoxia through negatively regulating the processing of the pri‐miRNA‐17‐92a to their respective miRNAs in hypoxic condition.^39^

The growth of tumor mass is dependent on its ability to respond to the hypoxic condition by inducing the expression of glucose transporters, glycolytic enzymes and vascular growth factors that enable adaptation and further tumor growth. In the light of evidence that Snd1 is overexpressed in several cancer types, it is highly relevant to investigate the effect and mechanisms of Snd1 and its overexpression on hypoxia response and cancer. Taken together, our results indicate an evolutionarily conserved role for Snd1 in hypoxia response in attenuating the hypoxia‐induced transcriptional response and regulating hypoxia adaptation.

The phenotype of the *Snd1* mouse knockout is rather subtle and may at first appear unexpected given the multiple functional assignments for the protein. However, our interpretation of the results is that the major function of Snd1 is a response function in reaction to external stimuli or stress, or during transformation. Several lines of evidence support this “response gene” concept. In mammalian cells, Snd1 has been shown to accumulate in cytoplasmic stress granules in response to various stress signals.^20^, ^21^ This is further supported by the BioID analysis that identified several stress granule proteins interacting with Snd1 (Figure 6). Similarly, to mammalian Snd1, the *A. thaliana* Snd1 was shown to localize in stress granules and processing bodies and *Snd1* deletion impaired tolerance to high salt concentration induced stress by downregulating mRNAs encoding secreted proteins.^22^, ^23^ Stress granules are formed of mRNA and stalled translation initiation complexes and may play a role in cellular response against harmful conditions. Additional evidence for Snd1 in stress responses has been provided in the context of breast cancer tumorigenesis where Snd1 stabilized the expression of pro‐survival genes under stress conditions through yet unknown mechanism.^16^ In this report, we provide further evidence for a role for Snd1 in stress in a physiological context, more precisely for proper hypoxia adaptation and attenuation of hypoxia‐responsive transcription.

Biotin Proximity Ligation Assay (BioID) was employed in normal and stress conditions to gain further insight into the Snd1 function. Most of the identified proteins (63 out of 96) were RNA‐binding proteins (Figure 6C). Most of the proteins in our analysis were putatively novel Snd1 interactors as only seven proteins (DDX3X, DHX9, EIF3CL, G3BP1, HNRNPU, MTDH and RPL23A) are listed in the BioGrid database as Snd1 interactors. Importantly, two of these proteins, G3BP1 and MTDH, are well‐established Snd1 interactors^16^, ^20^ supporting the validity of our analysis. Based on the number and coverage of the identified peptides, one of the highest confidence Snd1 interactor was the Microtubule Associated Protein 4 (MAP4), and also several other microtubule associated factors were identified (Figure 6E). Microtubules have also been previously shown to associate with Snd1 and this interaction could have a role in Snd1 trafficking and localization to stress granules.^40^ Another protein that was highly enriched in the analysis was SERBP1 (Serpine1 mRNA‐Binding Protein 1). Interestingly, the closest *Drosophila* homolog of the mammalian SERBP1 is VIG that is shown to interact with the fly Tudor‐SN in the RNA‐induced silencing complex (RISC).^6^ Two of the identified interactors, Trinucleotide repeat‐containing gene 6A and ‐ 6B (TNRC6A and TNRC6B) are components of the GW182 complex with a regulatory role in RNA interference in the RISC‐complex. Among the identified proteins were stress granule proteins including G3BP1, UBAP2L, CSDE1, and PRRC2C that are required for efficient formation of stress granules.^36^

*Snd1* gene ortholog is found in the fission yeast *Schizosaccharomyces pombe* but is lacking in the *Saccharomyces cerevisiae*. This expression pattern correlates with the more developed processes of gene regulation, pre‐mRNA splicing and RNA interference in *S. pombe* as compared to *S. cerevisiae* and it is consistent with the proposed roles for Snd1 in RNA metabolism. We investigated the function of Snd1 in mouse and show that in normal living conditions Snd1 has a rather subtle and organ/cell type specific effects on transcription. The results of this study link Snd1 to stress response and hypoxia related gene expression through miRNOme regulation.

## AUTHOR CONTRIBUTIONS

J. Saarikettu, M. Pesu, I. Junttila, J. Partanen, P. Sipilä, M. Poutanen, J. Yang, and O. Silvennoinen designed research; J. Saarikettu and S. Lehmusvaara performed research; J. Saarikettu and S. Lehmusvaara analyzed data; M. Pesu, I. Junttila, and J. Partanen contributed new reagents; and J. Saarikettu and O. Silvennoinen wrote the paper.

## FUNDING INFORMATION

This work was supported by the Academy of Finland (O.S 287573, M.P. 128623, M.P. 135980 and I.J. 25013080481), the Sigrid Juselius Foundation (O.S. and M.P.), the Finnish Cancer Foundation, Jane and Aatos Erkko Foundation, Tampere Tuberculosis Foundation and the Finnish Cultural Foundation, a Marie Curie International Reintegration Grant within the 7th European Community Framework Programme (MP), Emil Aaltonen Foundation (MP), and Competitive Research Funding of the Tampere University Hospital (OS grant 9V061, MP grants 9M080, 9N056, IJ 9N018), Competitive Research Funding of the Fimlab laboratories (IJ X51409) and Finnish Medical Foundation (IJ).

## DISCLOSURES

The authors declare no conflict of interest.

## Supporting information


**Appendix S1:** Supporting Information

## Data Availability

The gene expression data in this publication has been deposited in NCBI's GeneExpression Omnibus database (https://www.ncbi.nlm.nih.gov/geo/) and is accessible through GEO Series accession numbers GSE144217 for the spleen array data, GSE144060 for the liver array data, and GSE144309 for the liver small‐RNA seq data.
