# Supplementary material for: The RNA‐binding protein Snd1/Tudor‐SN regulates hypoxia‐responsive gene expression
Source: FASEB Bioadv. 2023 Feb 21;5(5):183–98. doi: 10.1096/fba.2022-00115 (PMC10158624; doi:10.1096/fba.2022-00115)
Supplement: Supplementary file 1 — Appendix S1: Supporting Information [file FBA2-5-183-s001.pdf]

SUPPORTING INFORMATION

SUPPLEMENTARY TABLE S1

Primers used for cloning of the *Snd1* targeting construct.

Primer name	Sequence
**Retrieval 5'1**	ATAAGCGGCCGCTGACAAGACGGAGCCTCTAAA
**Retrieval 5'2**	GTCAAGCTTGGCTTGTGAGCACTGTTGAG
**Retrieval 3'1**	GTCAAGCTTCTGTGCTGGGCTTTGTGATA
**Retrieval 3'2**	TCTACTAGTACTTCCCTCTGGTTGGCTCT
**NeoFloxP 5'1**	CGCGTCGACGCATGTTCATGCTCCACGTA
**NeoFloxP 5'2**	CGCGAATTCAAACAATTTTGAGGGGTTGC
**NeoFloxP 3'1**	CGCGGATCCATGCTGACCCCAAAACTTGA
**NeoFloxP 3'2**	CGCGCGGCCGCCTCCTGCTCCAACTGAGGTT
**NeoFRTloxP 5'1**	CGCGTCGACCAGGGTCCGTGTTTTCTCTC
**NeoFRTloxP 5'2**	CGCGAATTCGGGGATTGTTATCCGTGAAA
**NeoFRTloxP 3'1**	CGCGGATCCAAGCTTCAGCGGTCACTGTCACATTT
**NeoFRTloxP 3'2**	CGCGCGGCCGCCAGCAACCAACAAGTCATGG

SUPPLEMENTARY TABLE S2

Primers used in qRT-PCR gene expression analysis.

Target	Forward and reverse primer	Product size
Mouse Serpina1b	TGAGGCAGTGAAAGAACTGG<br>TCTCAGGATCGAATGGCTTC	95 bp
Mouse Camp	TCTACCGTCTCCTGGACCTG<br>CTCTGCCTTGCCACATACAG	107 bp
Mouse Ngp	GCCACTCCGCCTTCTAGTC<br>CTGTCTCTCCTGGGTGGAAG	93 bp
Mouse Mpo	CCTTCTTCACTGGCCTCAAC<br>GGTTCTTGATTCGAGGGTCA	96 bp
Mouse Ela2	CTTCTCTGTGCAGCGGATCT<br>ACGTTGGCGTTAATGGTAGC	105 bp
Mouse Prtn3	CAATTACAACCCCGAGGAGA<br>GACAGAGTCTGGTCCTGCTG	117 bp
Mouse Snd1	CTTTTCCGAGCGTACCTGTG<br>TCTGCAGCTAGCAGCTCATC	147 bp
Mouse Tbp	GCCTTCCACCTTATGCTCAG<br>TGCTGCTGTCTTTGTTGCTC	163 bp

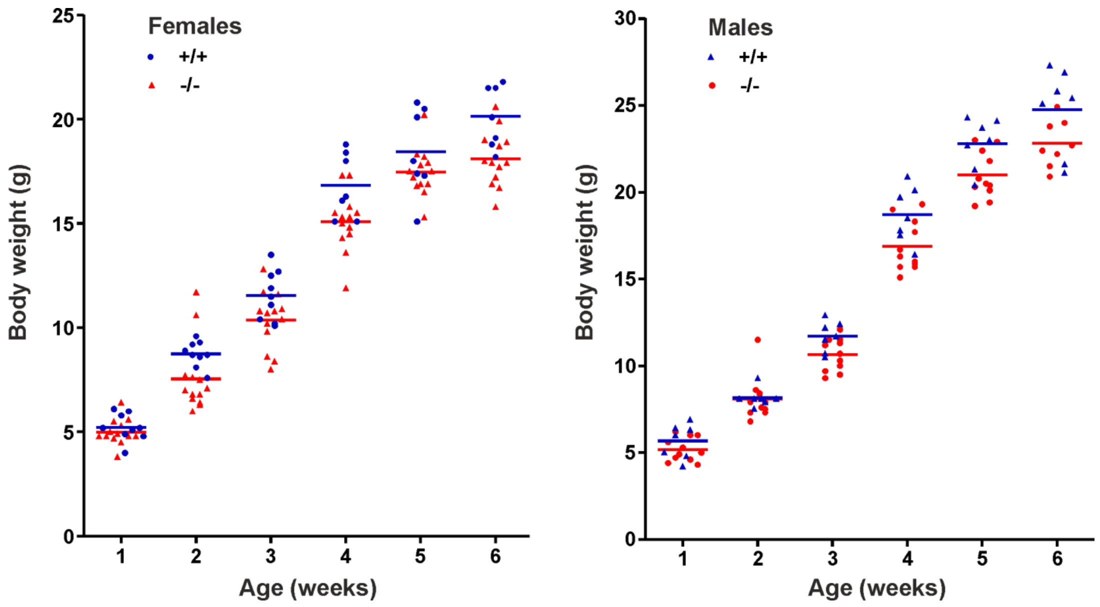

SUPPLEMENTARY FIGURE S1. Weight gain of the *Snd1* KO and WT mice. Weight of the animals was monitored from the first week after birth until week 6 of age.

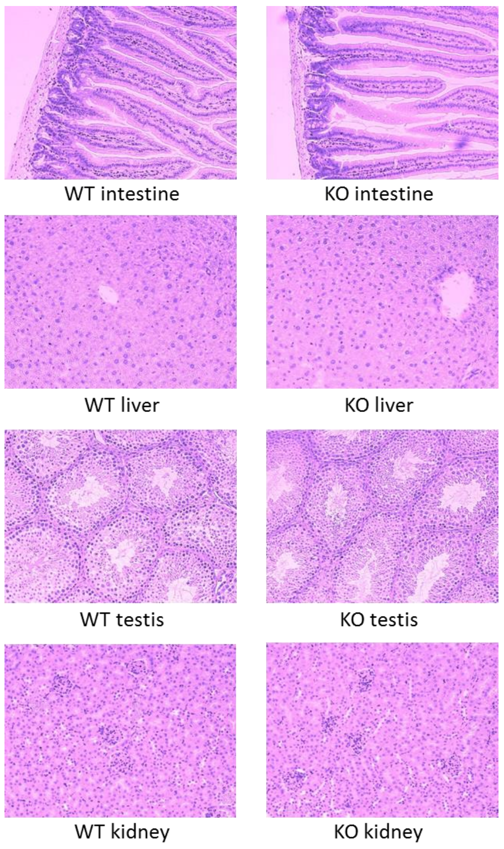

SUPPLEMENTARY FIGURE S2. Histological analysis of Snd1 KO mice. Paraffin embedded formalin fixed tissue sections from 2-moths old males were stained with hematoxylin and eosin. No evident changes were observed in the histology analysis.

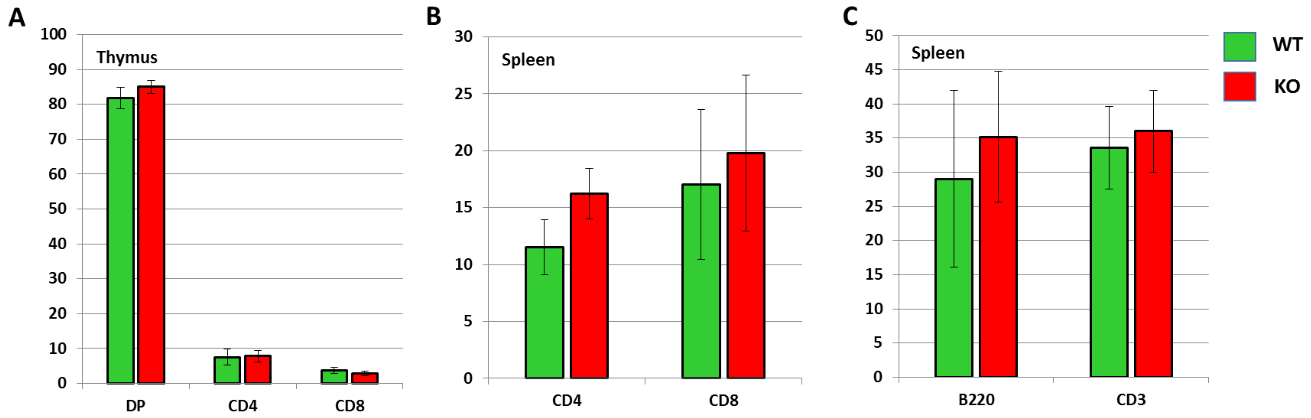

SUPPLEMENTARY FIGURE S3. FACS analysis of lymphocyte cell populations. A) CD4 and CD8 positive T-cells of thymus in WT and KO animals (n=6). DP = double positive. **B)** CD4 and CD8 positive T-cells of spleen in WT and KO animals (n=8) **C)** B220 positive B-cells and CD3 positive T-cells of spleen in WT and KO animals (n=8). Bars indicate the percentage of each cell population within the lymphocyte gate.

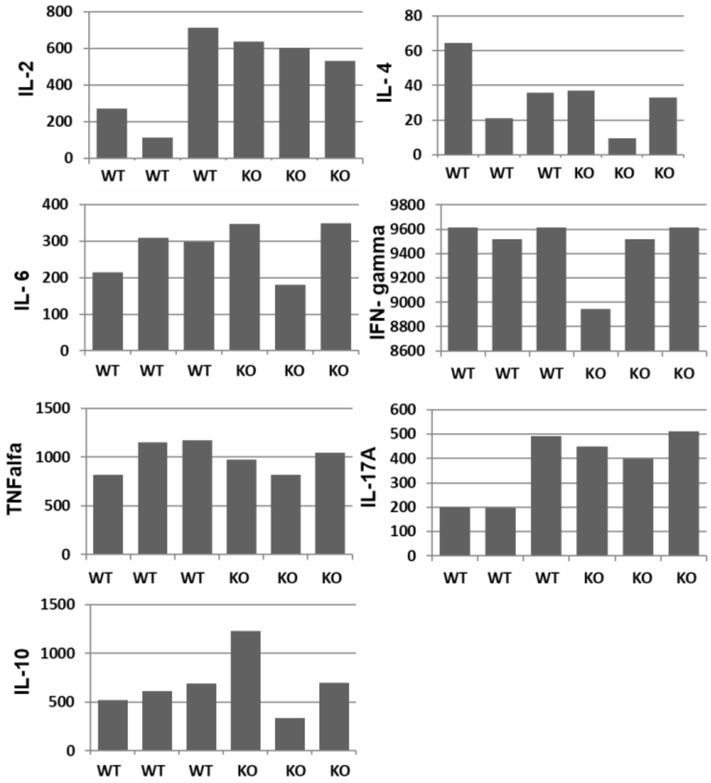

SUPPLEMENTARY FIGURE S4. Cytokine expression in cultured T-cells is not significantly affected in the *Snd1* knockdown. Splenocytes were cultured for 2 days on anti-CD3+anti-CD28 coated plates and the medium used for quantification of the cytokines (pg/ml). IL-2 was quantified using ELISA assay (Ebiosciences) whereas the other cytokines were determined with Mouse Th1/Th2/Th17 Cytokine Bead Array (BD Biosciences). Splenocytes for this analysis were from *Snd1* knockout (KO) animals (n=3) that were generated by breeding the *Snd1* Flox/Flox mice to PKG-Cre mice ubiquitously expressing CRE-recombinase. SND1 protein level in the expanded T-cells was reduced by >90 % compared to control cells (*Snd1* Flox/Flox without CRE, denoted here as WT) as assessed by Western Blot.

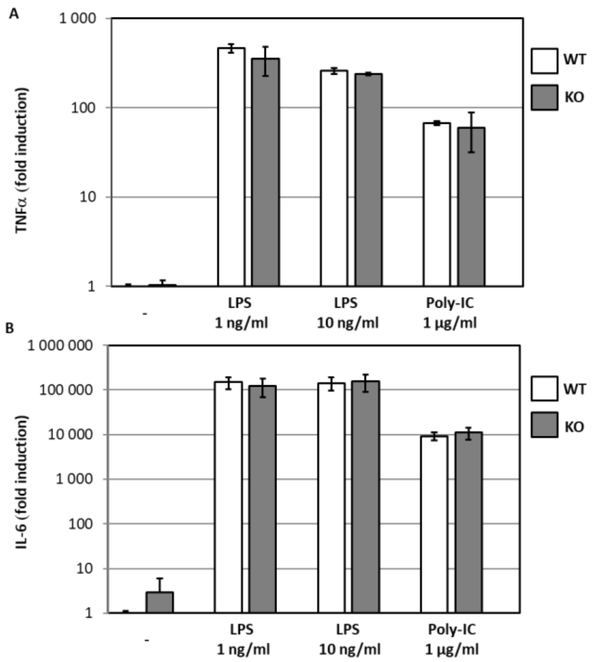

SUPPLEMENTARY FIGURE S5. Bone marrow derived macrophage activation is not affected in the KO. Bone marrow cells from WT and *Snd1* KO mice were cultured for 8 days in the presence of M-CSF (Peprotech). Cells were stimulated, where indicated, by Lipopolysaccharide (LPS, Sigma-Aldrich) or Poly-IC (Invivogen), for 3 hours. Expression of *TNFα* (**A**) and *IL-6* (**B**) was determined by qRT-PCR analysis by normalization to *Tbp* expression. Two individual 12-weeks old WT and KO male mice were used. Shown is the fold induction of expression, the average in unstimulated WT cells set as 1.

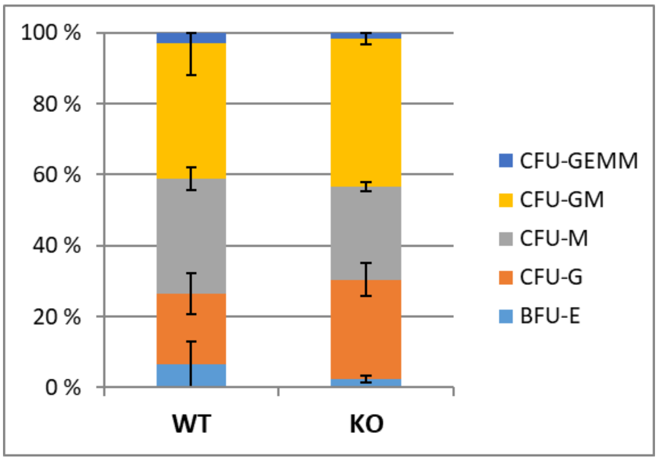

SUPPLEMENTARY FIGURE S6. Hematopoiesis is not significantly affected in the Snd1 KO in colony forming cell assay. The colony forming assay is based on the ability of hematopoietic progenitors to proliferate and differentiate as colonies in a semisolid medium that has been supplemented with cytokines to enable hematopoiesis. The colonies can be quantified and characterized according to their unique morphology. The bone marrow cells were isolated from 12-week-old WT and *Snd1* KO mice and grown in Methylcellulose medium (R&D systems, HSC007) according to manufacturer's instructions. The medium contains the cytokines to support all hematopoiesis except B cells. The cell colonies were identified after 8-10 days of culture by visual inspection under microscope from triplicate samples from three individual animals of both genotypes. Error bars represent standard deviation. CFU-GEMM, colony forming units generating myeloid cells; GM, granulocyte-macrophage progenitors; M, macrophages; G, granulocytes; BFU-E, burst forming unit erythroid.

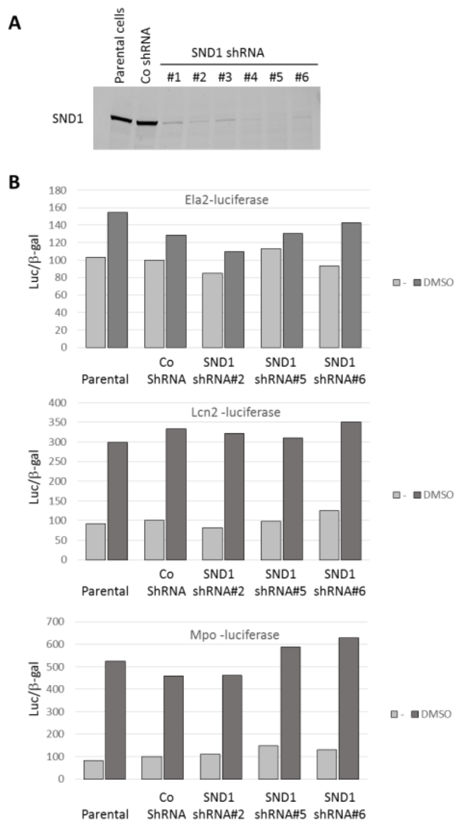

SUPPLEMENTARY FIGURE S7. SND1 silencing has no significant effect on the Ela2-, Lcn2- and Mpo-luciferase activities. A. Western Blot analysis of SND1 expression in lentiviral shRNA silenced K562 cells. **B.** *Ela2*-, *Lcn2*- and *Mpo*-luciferase activities in the SND1 silenced K562 cells in the absence or presence of DMSO for induction of myeloid differentiation.

RNAi Consortium shRNA library (Broad Institute, Cambridge, MA) *SND1* clones were used to produce lentiviral shRNA particles for silencing of the human *SND1*. The following shRNAs were used: TRCN0000049656 (CGGGATCTCAAGTATACCATT), TRCN0000245142 (TCTCGTCTCAAACTCTATTTG) and TRCN0000245143 (GCTGATGATGCAGACGAATTT).

K562 cells were maintained in RPMI supplemented with 10% FBS and penicillin-streptomycin. After lentiviral infection the cells with stable lentiviral integration were selected using 1 µg/ml Puromycin. For transient luciferase reporter transfections the K562 cells were electroporated using 5 µg of the luciferase reporters and 5 µg of CMV-b-gal for normalization. Electroporation was carried out using Bio-Rad Gene Pulser II Electroporation System with 340 volts and 975 µF. Cells were grown for 24 hours in the presence or absence of 1% DMSO for induction of myeloid differentiation before assaying the luciferase activity (Luciferase Assay System, Promega) and beta-galactosidase activity with 2-nitrophenyl-β-d-galactopyranoside as substrate and measuring the absorbance at 405 nm.

The promoter regions of mouse Ela2, Lcn2 and Mpo were PCR amplified using mouse genomic DNA as template. The following primer pairs were used: GCGAGATCTCTGAGGGGCACACATCTCTCC and GCGAAGCTTCCCCACATCCCTACCTAACAG for the ELA2 promoter (-133 to +1 with respect to the transcription start site), CACATCAGAAGAGGGTGTCAGATC and GCGAAGCTTCTCCCCATCCACCTGTCTTAAC for the Lcn2 promoter (-198 to +65) and GCGAGATCTGGGATCTGTTTCCCAGGTGATA and GCGAAGCTTAGGGTCACAGGGAAGATGTAGC for the Mpo promoter (-493 to +78). The amplified promoter sequences were cleaved and inserted into BglII and HindIII in pGL4.23 luciferase vector (Promega) that contains the cloning site followed by a minimal promoter and luciferase gene.

SUPPLEMENTARY TABLE S3. The expression values of 101 common genes between microarray and RNAseq analyses, in which expression was significantly altered in the SND1 KO mouse livers as compared to WT.

	gene expression microarray										RNAseq										
Gene	WT1	WT2	WT3	WT4	KO1	KO2	KO3	KO4	p.adjustec	logFC	WT1	WT2	WT3	WT4	KO1	KO2	KO3	KO4	logFC	PValue	FDR
Acot3	6.58	6.63	6.57	6.51	7.12	7.24	7.48	7.48	0.009405	0.76	12	16	6	6	34	92	94	44	2.47	1.15E-07	2.22E-05
Acot4	6.97	6.82	6.93	6.89	7.22	7.38	7.51	7.82	0.042355	0.58	598	702	615	665	960	1186	1546	1094	0.67	3.05E-06	0.000345
Acpp	7.07	7.08	6.89	6.83	6.61	6.55	6.63	6.59	0.030059	-0.37	341	249	123	273	117	31	111	152	-1.46	0.001265	0.031365
Acsl3	7.58	7.55	7.54	7.49	7.87	8.27	8.54	8.59	0.034964	0.78	887	897	926	940	1370	2124	2317	1587	0.79	3.98E-07	6.53E-05
Agxt	10.35	10.21	10.17	10.04	10.59	11.04	11.49	11.12	0.040805	0.87	7207	8668	6409	8572	11401	18543	26465	11695	0.92	0.000162	0.00772
Amigo2	7.1	7.25	7.16	7.26	7.63	7.36	7.55	7.62	0.044183	0.35	100	110	144	107	267	187	206	205	0.69	0.001131	0.029171
Apcs	8.59	9.24	8.92	8.84	7.34	8.07	7.97	7.63	0.025819	-1.15	5549	3898	12670	3274	1833	1880	1749	1026	-2.22	2.21E-07	3.89E-05
Apon	8.78	8.83	8.65	8.77	8.37	8.33	8.52	8.42	0.028665	-0.35	7109	7859	8124	7763	6438	5361	5924	5862	-0.60	4.32E-06	0.000445
Aqp8	8.62	8.67	8.94	8.71	7.71	7.01	7.55	7.31	0.008278	-1.34	1640	2394	1051	2316	518	318	804	1061	-1.64	7.98E-06	0.000727
Arf5	8.33	8.34	8.17	8.19	7.88	7.73	7.84	7.86	0.016235	-0.43	563	594	591	680	469	392	402	304	-0.84	1.95E-08	5.21E-06
Ass1	12.68	12.37	12.38	12.45	13.26	13.02	13.1	13.13	0.012811	0.66	47416	59592	41479	74867	78498	130552	135731	93918	0.76	0.000377	0.014163
Bok	8.01	8.32	8.22	8.2	7.49	7.66	7.53	7.73	0.016235	-0.59	403	375	249	411	277	227	250	246	-0.73	6.88E-05	0.003934
Cbr1	8.66	8.28	8.46	8.48	9.02	9.29	9.25	8.98	0.021171	0.66	694	1115	1286	1070	1705	2040	2202	1448	0.61	0.001437	0.034282
Cd74	8.54	8.45	8.64	8.76	9.2	9.2	9.06	9.04	0.018219	0.53	522	627	608	843	1092	1438	1303	768	0.61	0.001265	0.031365
Cpne8	7.32	7.72	7.16	7.41	6.79	6.88	6.69	6.62	0.030059	-0.66	255	180	473	236	189	129	90	147	-1.26	0.000359	0.013725
Cps1	12.75	12.43	12.26	12.16	12.91	13.19	13.31	13.46	0.034618	0.82	230110	255303	252763	348110	373142	492748	517935	435490	0.53	0.001546	0.03582
Csrp3	6.97	7.07	7.11	6.96	6.84	6.72	6.62	6.6	0.040805	-0.33	550	617	536	535	426	226	215	324	-1.11	3.25E-06	0.000354
Cyp17a1	7.68	7.29	7.17	7.65	8.04	8.99	8.97	9.49	0.040805	1.43	137	117	393	232	519	1114	1028	698	1.70	1.23E-05	0.001025
Cyp2d12	6.6	6.81	6.67	6.69	6.46	6.45	6.44	6.46	0.048822	-0.24	41	12	22	15	7	2	3	10	-2.22	0.000325	0.013106
Cyp2f2	12.71	12.95	12.76	12.81	12.44	12.21	12.51	12.32	0.032846	-0.44	14895	20808	15194	19893	13605	10822	12784	15531	-0.62	0.000339	0.013405
Cyp4a14	7.04	6.49	6.62	6.45	10.06	9.11	9.1	8.16	0.017263	2.46	9	82	48	96	2442	2481	2241	381	4.80	2.46E-11	1.43E-08
Cyp4b1	7.11	7.1	7.22	7.06	7.35	7.39	7.52	7.66	0.046883	0.36	697	901	455	835	1079	1473	1682	1177	0.69	0.000512	0.017686
Cyp7b1	12.48	12.94	12.86	12.61	11.35	10.79	10.75	10.78	0.003008	-1.81	18894	19635	17800	15296	10348	3183	2729	10903	-1.59	0.000449	0.016389
Cyp8b1	12.37	12.38	12.63	12.51	11.66	9.69	10.05	10.72	0.038589	-1.94	8777	13437	6376	13415	5817	1195	1595	5770	-1.71	0.000925	0.025598
Dct	8.58	8.74	8.84	8.51	6.97	6.81	6.92	6.74	0.000054	-1.81	275	298	147	201	91	23	65	63	-2.13	5.15E-08	1.12E-05
Dhx58	7.36	7.47	7.35	7.5	7.21	7.08	7.14	7.05	0.035577	-0.30	352	428	364	481	385	283	366	311	-0.48	0.002076	0.044034
Dio1	8.47	9.2	9.4	9.42	8.13	6.77	7.12	7.22	0.029688	-1.81	3976	6283	4605	4721	1856	240	459	2480	-2.13	0.000649	0.020832
Dnajb2	8.92	8.76	8.64	8.9	9.13	9.4	9.14	9.35	0.040805	0.45	870	1059	976	1208	1428	1946	1641	1452	0.44	0.000767	0.022737
Ear2	7.28	7.47	7.6	7.56	6.83	6.94	6.91	6.75	0.012811	-0.62	110	134	95	97	45	40	65	35	-1.46	4.33E-09	1.4E-06
Elovl1	7.73	7.73	7.61	7.74	8.39	8.04	8.1	8.06	0.033863	0.44	780	728	813	750	1253	1238	1434	1197	0.52	1.53E-05	0.001218
Elovl3	7.9	7.79	8.13	7.79	7.36	7	6.82	7.12	0.020483	-0.83	2831	5255	1647	2583	1848	1137	594	1263	-1.54	0.000104	0.005474
F2r	9.96	10.6	10.56	10.42	9.73	9.22	9.7	9.72	0.047719	-0.79	1901	1920	1782	1799	1183	726	804	1293	-1.09	3.13E-07	5.35E-05
Fads1	12.47	12.3	12.18	12.32	12.71	13.04	12.99	13.33	0.030059	0.70	6779	8446	7501	8323	17783	19819	18140	16100	1.00	5.91E-15	6.43E-12
Fads2	8.7	8.34	8.4	8.59	9.53	9.49	9.47	10.04	0.012811	1.13	6983	8291	6749	9499	24350	32236	29064	22932	1.57	6.48E-25	1.61E-21
Fbp1	12.06	11.78	11.84	11.99	12.21	12.34	12.45	12.46	0.03326	0.45	19665	25058	17918	30470	32394	42139	45235	36052	0.54	0.002081	0.044083
Fbxo6	10.04	10.33	10.29	10.25	9.59	9.56	9.49	9.55	0.005276	-0.68	996	1008	842	860	761	627	684	674	-0.65	3.08E-06	0.000345
Fh1	10.51	10.11	10.17	10.23	11.1	11.03	11.23	11.2	0.006462	0.89	5509	5758	5967	8883	9851	15406	13393	10191	0.69	0.000185	0.008605
Fkbp4	10.43	10.41	10.41	10.62	10.91	10.85	10.84	10.84	0.019419	0.39	4161	5125	4343	7096	7462	10138	8657	7896	0.52	0.002066	0.043978
Gale	7.3	7.07	6.93	7.2	7.52	7.86	7.58	7.55	0.04266	0.50	379	447	579	470	1121	2305	1368	930	1.38	1.54E-07	2.84E-05
Gck	9.26	8.58	8.28	9.02	9.98	10.02	9.8	9.91	0.028665	1.14	3050	2460	3424	5261	12733	14376	12172	12063	1.65	3.59E-15	4.17E-12
Gna14	7.13	7.5	7.28	7.61	6.7	6.56	6.67	6.67	0.016235	-0.73	309	253	194	363	91	48	55	207	-1.65	0.000274	0.011405
Gsta2	7.08	6.75	6.92	6.75	7.27	7.91	7.96	8.22	0.040196	0.97	303	364	201	528	726	1540	2267	1057	1.79	1.57E-07	2.88E-05
Gsta4	8.25	8.07	7.99	7.94	8.66	9	8.78	8.69	0.013214	0.72	801	861	634	1041	1325	2311	2737	1720	1.06	3.32E-07	5.62E-05
Gstm1	11.31	11.18	11.12	10.83	11.61	11.66	11.88	11.78	0.028774	0.62	5181	6654	4953	6803	10433	10807	14044	7427	0.64	0.000326	0.013106
Gstp1	12.83	13.13	13.1	12.95	12.17	12.47	12.19	11.92	0.018219	-0.81	19988	19307	20776	20446	18295	10248	10967	12689	-0.83	6.06E-05	0.00359
Gstp2	7.53	8.05	7.72	7.35	7.02	6.87	6.78	6.78	0.029905	-0.80	64	61	358	46	42	28	19	41	-2.26	0.000955	0.026214
Gstt2	8.51	8.51	8.74	8.57	9.54	9.34	9.15	9.01	0.028192	0.68	1380	1580	1023	1586	2436	2389	2982	2222	0.64	1.52E-05	0.001215
H2-Ab1	7.64	7.38	7.46	7.67	8.04	7.92	8.12	8.13	0.024604	0.52	277	475	432	507	747	823	868	534	0.60	0.002086	0.044116
Hsd17b2	10.43	10.96	10.94	10.54	9.91	9.83	9.69	9.49	0.018793	-0.99	6776	7854	6511	6187	5109	3813	4272	3835	-0.90	5.5E-09	1.67E-06
Hsd3b5	11.47	12.08	12.14	11.61	10.27	10.51	10.94	10.57	0.020671	-1.25	13421	14450	7387	8820	8572	4310	5995	7042	-0.97	0.000505	0.017561
Ifi47	7.65	7.64	7.77	7.63	7.02	7.28	7.26	7.32	0.025819	-0.45	555	654	739	506	447	413	441	379	-0.77	5.07E-06	0.00051
Igfbp2	9.44	9.58	9.55	9.56	10.57	10.02	10.06	10.25	0.027525	0.69	7211	8567	4508	7367	18563	16313	14626	10234	0.90	5.62E-05	0.00341
Ilvbl	8.01	7.92	7.95	7.81	8.22	8.25	8.39	8.37	0.025819	0.39	581	673	527	729	854	1140	1045	839	0.41	0.002361	0.047967
Inmt	11.94	11.54	11.65	11.77	12.34	12.61	12.88	12.77	0.016235	0.93	10426	11649	3831	16227	20196	29852	43514	33658	1.40	0.000129	0.006529
Insig1	8.1	8.27	8.29	8.25	8.6	8.82	9.43	9.19	0.047831	0.78	9226	8979	7545	8737	13783	35171	33553	18329	1.32	9.94E-07	0.000142
Insig2	8.18	7.48	7.65	7.6	7.09	6.72	6.7	6.63	0.029414	-0.94	2275	3401	2483	2316	1585	1685	1369	2034	-0.86	5.84E-06	0.000561
Irf5	7.27	7.36	7.51	7.22	7.11	7.01	6.94	6.94	0.049452	-0.34	616	575	674	620	578	443	556	364	-0.58	0.000577	0.019148
Lcn2	10.18	11.04	9.95	11.58	8.54	6.98	6.92	6.94	0.016235	-3.34	1062	452	10447	890	302	39	19	46	-5.22	2.52E-05	0.00178
Ldhd	7.8	8.03	7.94	7.95	7.73	7.51	7.6	7.61	0.045103	-0.32	5081	5259	5135	5484	4808	4281	3905	4065	-0.51	6.59E-05	0.003848
Ldlr	6.96	7.15	7.04	7.13	7.36	7.34	7.4	7.57	0.040805	0.35	8024	7636	5498	7958	11699	16009	15421	14089	0.76	2E-06	0.000246
Lonp2	10.88	10.96	11.23	10.84	10.27	10.42	10.46	10.69	0.049114	-0.52	14237	18160	11328	14847	13083	10381	10542	12403	-0.54	0.002072	0.044034
Ly6a	8.03	8.26	8.13	8.62	7.66	7.52	7.33	7.2	0.029688	-0.83	147	113	202	167	88	62	72	74	-1.30	1.99E-07	3.53E-05
Ly6e	8.56	9.45	8.85	9	8.15	8.15	8.13	8.28	0.04574	-0.79	3038	2591	3679	2988	1869	1533	1784	1252	-1.16	2.21E-11	1.37E-08
Mmrn2	7.67	7.54	7.63	7.75	8.06	8.02	7.95	7.95	0.025819	0.35	440	519	485	661	836	971	1000	722	0.53	0.000191	0.008868
Moxd1	8.11	11.26	10.97	10.7	6.48	6.5	6.49	6.38	0.025953	-3.80	10439	8589	7922	8394	68	14	10	48	-8.16	4.81E-36	1.67E-32
Nhlrc2	7.75	7.61	7.81	7.69	8.07	8.04	8.12	8.2	0.020483	0.39	701	906	816	1146	1371	1362	1427	1438	0.45	0.001179	0.029884
Nr1d1	7.32	7.47	7.26	7.06	6.98	6.78	6.72	6.75	0.042053	-0.47	880	764	647	585	692	306	341	196	-1.13	0.001089	0.028544
Nudt19	8.09	7.98	7.73	8.06	8.34	8.39	8.7	8.77	0.044183	0.58	857	957	835	1238	1438	1908	2034	1535	0.62	1.57E-05	0.001238
Odc1	9.55	9.21	9.32	9.51	9.78	10.11	10.02	9.91	0.02974	0.56	1790	2002	2302	2289	4109	4627	4092	3808	0.77	1.67E-09	6.18E-07
Orai1	7.71	7.61	7.55	7.59	7.99	8.01	7.88	7.8	0.04266	0.31	123	183	127	172	266	316	331	187	0.65	0.001677	0.038115
Orm2	10.07	11.75	10.64	12.04	9.57	8.6	8.15	7.79	0.040805	-2.60	3054	1295	9509	2024	772	171	169	164	-3.87	5.51E-07	8.71E-05
Papss1	7.13	7.28	7.28	7.25	8.14	8.3	8.35	7.58	0.03326	0.86	202	188	129	181	492	749	651	383	1.48	3.59E-11	1.9E-08
Pcsk9	9.15	9.16	9.01	9.15	9.89	10.03	9.78	9.95	0.002565	0.79	1572	1868	1525	1301	4199	5326	4315	3825	1.27	1.38E-15	1.85E-12
Pdzrn3	7.09	7.32	7.18	7.11	6.62	6.88	6.76	6.74	0.028665	-0.42	174	195	297	184	26	80	55	106	-1.88	1.98E-06	0.000245
Ppl	7.28	7.17	6.9	7.13	8.06	8.24	8.03	8.2	0.003008	1.01	741	470	1545	930	1967	2817	2487	1505	1.01	0.002385	0.048131
Prodh2	10.57	10.51	10.49	10.43	10.87	10.73	10.88	10.82	0.025819	0.33	5464	5923	4412	6908	7576	9307	10132	8894	0.45	0.001972	0.042436
Prss8	6.75	7.08	6.94	6.94	7.19	7.37	7.53	7.61	0.04574	0.50	175	174	93	163	269	491	608	311	1.25	8.5E-06	0.000766
Psmb9	7.87	8.39	8.33	8.46	7.52	7.72	7.73	7.73	0.049987	-0.59	521	479	506	586	404	372	401	469	-0.55	0.000151	0.007315
Qsox1	8.79	9	9.02	9.03	8.67	8.4	8.55	8.41	0.03117	-0.45	15971	19480	20799	17337	16404	14215	14720	12274	-0.57	0.00023	0.010155
Rdh11	7.79	7.67	7.89	7.74	8.05	8.19	8.38	8.58	0.047719	0.53	1623	1953	1569	1503	3048	3589	3695	3041	0.79	1.85E-09	6.59E-07
Rexo2	8.03	8.04	7.93	8.14	7.83	7.6	7.75	7.57	0.046883	-0.35	920	1000	964	1034	868	886	838	933	-0.36	0.001718	0.038494
Rnf145	7.51	7.42	7.31	7.39	8	8.32	8.2	8.2	0.005189	0.77	569	502	467	650	1236	1250	1330	890	0.89	2.68E-09	9.32E-07
Rorc	6.73	6.75	6.77	6.75	7.09	6.87	7.09	7.11	0.044841	0.29	1502	1617	1004	2689	2981	2696	4209	6622	1.10	0.001132	0.029171
Saa1	9.16	10.01	9.78	10.19	8.73	7.18	6.64	6.73	0.033863	-2.47	23058	22037	71322	24749	10490	1334	537	1564	-3.55	5.33E-05	0.00329
Saa2	9.54	9.33	9.04	9.99	8.3	6.81	6.58	6.58	0.025953	-2.41	15980	13970	72509	15394	5361	390	262	701	-4.35	2.95E-05	0.002039
Selenbp2	11.21	11.93	11.75	11.78	10.53	9.98	10.19	10.12	0.009753	-1.46	69674	66715	46308	79464	33197	14872	18446	30145	-1.63	2.54E-08	6.48E-06
Serpina6	9.18	9.26	9.43	9.28	10.2	10.46	10.77	11.1	0.01311	1.35	1477	1732	1261	1993	2965	3990	5095	3597	1.06	6.21E-10	2.46E-07
Serpina7	8.23	7.99	7.89	7.48	8.68	8.72	8.63	8.82	0.02969	0.82	1207	947	709	732	1876	2803	2284	1295	0.97	0.000101	0.005337
Sigirr	7.03	7.22	7.17	7.17	7.37	7.44	7.41	7.48	0.038444	0.28	409	443	392	479	603	967	898	624	0.62	0.000154	0.007423
Slc22a7	7.61	7.91	7.89	7.86	7.47	6.92	6.89	6.91	0.031718	-0.77	1249	1785	1330	1499	687	188	134	1002	-1.71	0.002291	0.046928
Slc5a6	8.04	8.23	7.91	8.38	8.51	8.81	8.86	8.71	0.040805	0.58	323	279	316	399	622	683	628	407	0.61	0.000608	0.019813
Snd1	7.52	7.51	7.5	7.57	6.48	6.46	6.47	6.41	0.000015	-1.07	5476	5823	6206	6720	12	23	27	10	-8.60	0	0
Sptlc1	8.66	8.59	8.52	8.39	8.99	8.89	9.16	9.05	0.025819	0.48	606	688	665	696	1027	1217	1115	978	0.49	1.7E-05	0.001316
Stip1	8.96	8.43	8.45	8.75	9.77	9.43	9.41	9.15	0.040805	0.79	1065	1348	1661	1807	3122	3185	2766	2548	0.77	3.45E-06	0.000373
Sult5a1	7.02	7.31	7.43	7.08	6.75	6.7	6.63	6.56	0.02969	-0.55	568	794	510	566	412	171	128	274	-1.50	1.62E-05	0.001263
Tlcd2	8.22	8.45	8.67	8.33	7.75	7.42	7.5	7.74	0.016235	-0.82	3157	3735	3398	3363	2055	1238	1405	2646	-1.09	1.11E-05	0.000936
Tm6sf2	7.48	7.28	7.37	7.55	7.83	8.01	7.92	7.77	0.026528	0.46	308	352	464	508	699	924	1034	555	0.76	0.00032	0.013006
Ubxn1	10.32	10.29	10.25	10.25	9.74	9.71	9.7	9.6	0.002418	-0.59	1695	2062	1422	1788	1575	1498	1540	1243	-0.46	0.000545	0.018445
Usp2	8.17	8.69	8.18	8.79	9.49	9.35	9.36	9.79	0.025819	1.04	324	284	241	568	668	802	783	884	0.95	4.9E-05	0.003113
Wipi1	7.64	7.78	7.78	7.89	7.37	7.06	7.12	7.08	0.014614	-0.61	476	560	461	428	449	234	349	360	-0.68	0.001471	0.034884
Vldlr	6.53	6.46	6.49	6.53	6.82	7.01	6.82	6.97	0.016598	0.4025	11	16	30	34	81	276	200	91	2.60	4.91E-08	1.1E-05

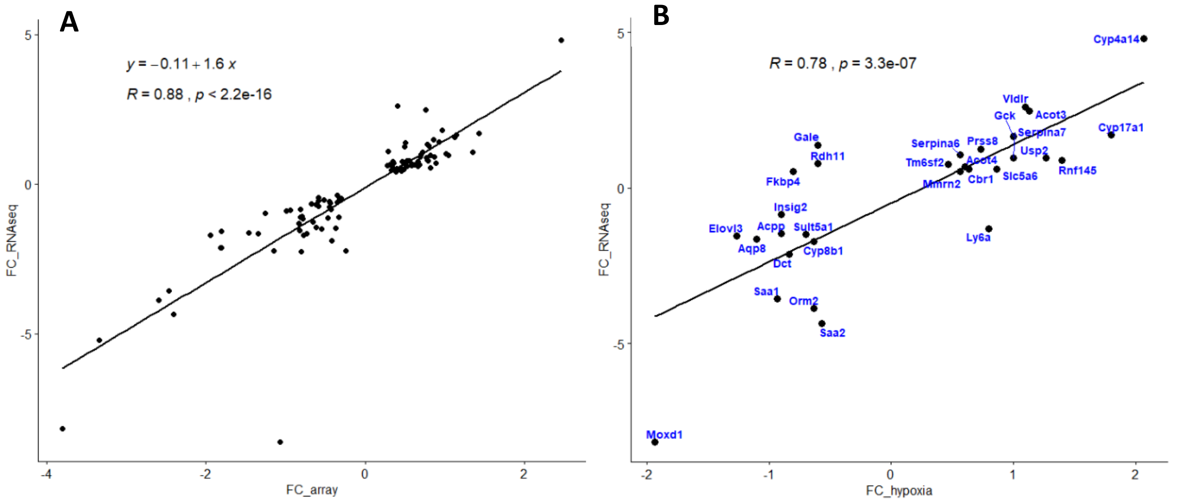

SUPPLEMENTARY FIGURE S8. Correlation of gene expression changes. **A.** Expression correlation of 101 genes in which expression was significantly altered in KO mouse livers in both microarray and RNA sequencing analysis. **B.** Correlation between *SND1* KO liver and adaptation to hypoxic environment. Figure shows genes which are common in microarray and RNA sequencing data analysis (Y axis) and detected also in hypoxic environment (X axis). Fold changes (FC) are presented as log2 values.

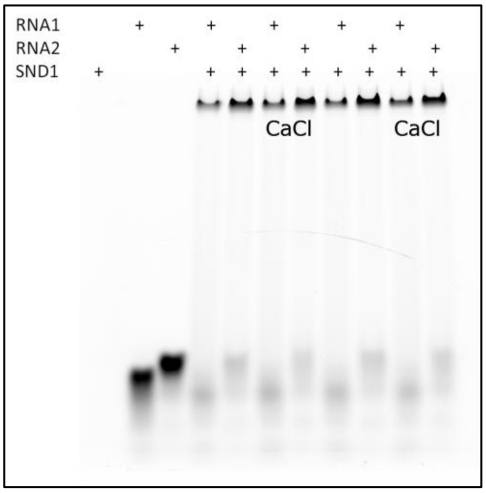

SUPPLEMENTARY FIGURE S9. Electrophoretic Mobility Shift Assay of miRNA binding by pure recombinant SND1 protein. Fluorescently labelled miRNAs were incubated with pure full-length SND1 protein in the presence of either 5 mM CaCl2 or 5 mM EDTA. After the binding reaction, the RNAs were resolved on a Polyacrylamide gel and the RNA were visualized by detecting the fluorescence. The SND1 protein production and Electrophoretic Mobility Shift Assay (EMSA) method has been described previously (1). The fluorescent RNA probes were 5' Alexa Fluor 488 labelled Hsa-miR-96-5p (UUUGGCACUAGCACAUUUUUGCU, RNA1) and 5' Alexa Fluor 488 labelled Hsa-miR-182-5p (UUUGGCAAUGGUAGAACUCACACU, RNA2) from Integrated DNA Technologies, Leuven, Belgium.

(1) Lehmusvaara, S., Haikarainen, T., Saarikettu, J., Martinez Nieto, G., and Silvennoinen, O. (2022) Inhibition of RNA Binding in SND1 Increases the Levels of miR-1-3p and Sensitizes Cancer Cells to Navitoclax. Cancers 14(13), 3100.
